# Self‐Assembly of a Colloidal Honeycomb Crystal in Silico for Topological Valley Photonics

**DOI:** 10.1002/smll.75515

**Published:** 2026-10-04

**Authors:** Adam Walker, Andreas Neophytou, Piet J. M. Swinkels, Angela Demetriadou, Peter Schall, Dwaipayan Chakrabarti

**Affiliations:** ^1^ EPSRC Centre for Doctoral Training in Topological Design University of Birmingham Birmingham United Kingdom; ^2^ School of Chemistry University of Birmingham Birmingham United Kingdom; ^3^ Dipartimento di Fisica Sapienza Università di Roma Roma Italy; ^4^ School of Physics and Astronomy University of Birmingham Birmingham UK; ^5^ Institute of Physics University of Amsterdam Amsterdam The Netherlands; ^6^ International Institute for Sustainability with Knotted Chiral Meta Matter (WPI‐SKCM^2^) Hiroshima University Hiroshima Japan

**Keywords:** colloidal photonic crystals, photonic insulator, self‐assembly, topological defect, waveguide

## Abstract

The quest to employ colloidal self‐assembly as a scalable means of fabricating photonic crystals with a complete photonic band gap at optical frequencies has proved challenging throughout the first quarter of the 21st century. Meanwhile, the field of topological photonics has emerged, presenting new, and largely unexploited, functionalities for self‐assembled colloidal materials. The robustness of topological photonic properties to defects that are practically unavoidable in self‐assembled colloidal structures appears to be especially attractive. Here, the self‐assembly of a colloidal honeycomb crystal is demonstrated in computer simulations, with an assembly strategy that not only suppresses the formation of real‐space topological defects observed in experiments but also results in topological photonic properties in reciprocal space. This assembly strategy promotes the emergence of long‐range order and allows for the breaking of spatial inversion symmetry with two distinct sub‐lattices. A sufficient dielectric contrast between the colloidal particles sitting on these two sub‐lattices is exploited to realize a valley‐Hall topological photonic phase, allowing for unidirectional propagation of circularly polarized light through waveguides composed of the designer crystals. The design rules are tested for their robustness to open the door to scalable fabrication of topological photonic devices via colloidal self‐assembly.

## Graphical abstract

The self‐assembly of a colloidal honeycomb crystal is demonstrated in computer simulations, using an assembly strategy that not only suppresses the formation of real‐space topological defects observed experimentally but also yields topological photonic properties in reciprocal space. The robustness of the design rules is envisaged to pave the way for scalable fabrication of topological photonic devices via colloidal self‐assembly.

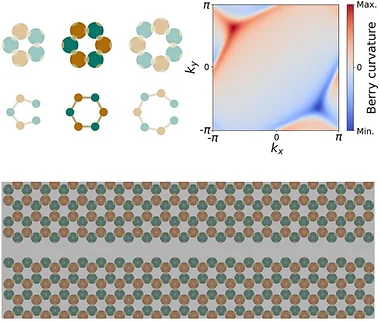

## 1 Introduction

Colloidal self‐assembly promises to offer a viable route for scalable and sustainable fabrication of photonic materials operating at optical frequencies [1, 2, 3]. Much of the focus in the field of colloidal self‐assembly – driven by remarkable advances in colloid synthesis through the first quarter of the 21st century [4, 5, 6, 7] – has been on realizing 3D photonic crystals with a complete photonic band gap at optical frequencies [8, 9, 10, 11]. Meanwhile, topological photonics has emerged as an exciting field of research of its own [12, 13, 14], motivated by the plethora of opportunities that synthetic optical materials offer for fundamental understanding of topological phenomena, as well as for their applications in photonics [13, 15, 16, 17, 18]. A remarkable feature of topological photonic properties is their robustness to disorder and imperfections [19]. This topological protection has huge potential for exploitation in the scalable fabrication of advanced materials via colloidal self‐assembly since defects are practically unavoidable in such a bottom‐up route [20]. Therefore, topological photonic materials operational at optical frequencies present attractive targets for programmable colloidal self‐assembly [21].

One of the most famous examples of materials with exciting properties of topological origin is graphene [22], comprised of carbon atoms arranged in a 2D honeycomb lattice. Over the last twenty years, honeycomb and related 2D lattices have emerged as a rich playground for topological physics in electron, photon, and phonon transports [23]. Central to these developments is the existence of two energetically degenerate points in the reciprocal space for a honeycomb lattice. These can be made inequivalent to provide an additional degree of freedom – the valley degree of freedom ‐ with rich topological physics, having applications in electronics, photonics and phononics [24, 25, 26]. In most realizations of valley topological photonic crystals [27], a geometric parameter is utilized to break the inversion symmetry in a honeycomb lattice [25]. Notably, topological protection against backscattering on structural defects has been called into question recently, based on measurements of losses in the slow‐light regime of valley‐Hall topological waveguides, which exploit the inversion symmetry breaking in a honeycomb structure [28].

In this work, we establish a two‐component colloidal self‐assembly strategy both as a bottom‐up route to the honeycomb crystal, and as a means to encode inversion symmetry breaking for valley photonics at optical frequencies. Although the self‐assembly of colloidal honeycomb – an archetypal 2D colloidal open crystal – has been considered in computational studies [30, 31], it has proven challenging in experiments [29, 32]. A recent experimental study has revealed the abundance of topological defects in the course of the self‐assembly of colloidal honeycomb from tetrahedral patchy particles on a substrate [29]. Alongside the ideal 6‐member rings, the prevalence of 5‐ and 7‐member rings as topological defects, evident in Figure 1a, is found to interrupt the emergence of long‐range order. To address this issue, which is illustrated schematically for a one‐component system in Figure 1b, we employ a two‐component strategy, where specific interactions between patches allow bonding only between different types of particles [33], as shown schematically in Figure 1c. We demonstrate in silico the self‐assembly of tetrahedral patchy particles on a substrate with this two‐component strategy, which promotes the emergence of long‐range order over its one‐component counterpart through suppression of topological defects. Moreover, this two‐component assembly strategy causes the two types of particles to sit on distinct sub‐lattices, resulting in the breaking of inversion symmetry. We show that a sufficient contrast in the dielectric constant between the two types of particles can then be exploited to give rise to the valley‐Hall topological photonic phase, thereby opening the door to scalable fabrication of topological photonic devices.

**FIGURE 1:**
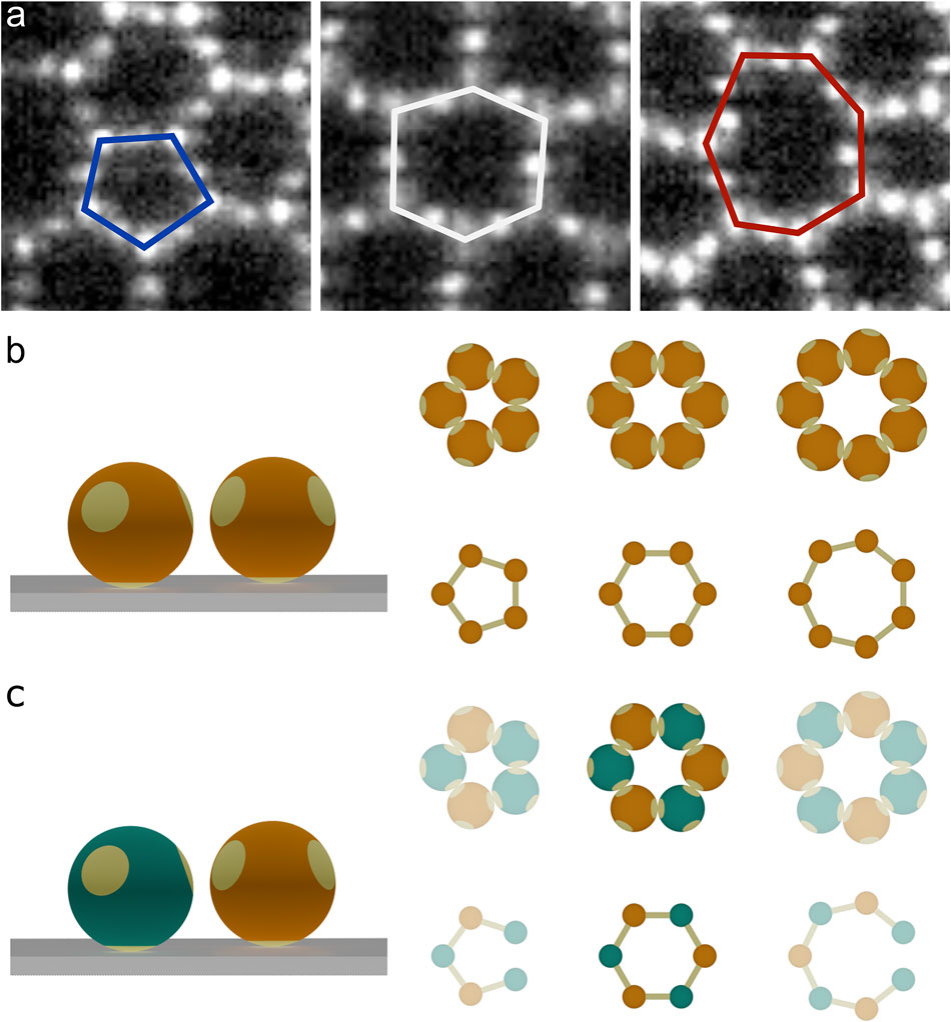
Suppression of topological defects via specific interactions between tetrahedral patchy particles in a 1:1 two‐component system on a substrate. (a) A one‐component system of tetrahedral patchy particles on a substrate forms topological defects (5‐member and 7‐member rings), alongside ideal structural motifs (6‐member rings), as reported in a recent experimental study [29]. The confocal microscope images highlight the fluorescent patches of the particles, allowing reconstruction of their bonding topology (see superimposed pentagon, hexagon and heptagon). (b,c) Schematics illustrating how both odd‐ and even‐member rings can be formed in a one‐component system (b), but the topological defects can be suppressed in a two‐component system by exploiting specific interactions between the patches (c). In the two‐component system, only the patches on two different types of particles (having distinct colors) can interact attractively to give rise to “bonds”, which we represent as edges between nodes (located at particle centers). In both systems, the tetrahedral patchy particles can bind to the surface through one of their patches.

## 2 Results

### 2.1 Self‐Assembly of Colloidal Honeycomb

We first report the self‐assembly of tetrahedral patchy particles on a two‐dimensional substrate in two different systems. Each patchy particle is spherical in shape and has four circular patches in a tetrahedral arrangement, but the energetics of the interactions bias the particles to remain anchored near the substrate, engaging one of the patches. Here, each particle is thus pseudo‐trivalent. We consider a one‐component system and a two‐component system with a 1:1 mixture. The only attractive interactions between the particles in the latter system are between the patches on the two different types of particles (see Figure 1c). Such a specificity of interactions can be realized using DNA functionalized patches [7, 34, 35]. The effective interactions between the patches are described by the widely used Kern–Frenkel potential [36]. Both systems are subject to gradual cooling in Monte Carlo simulations. See Methods for a detailed description of the potential and the simulation procedure followed.

Figure 2a,d shows the average energy per particle as a function of the reduced temperature, $T^{*}$, for the one‐component and two‐component systems, respectively. With falling $T^{*}$, a decrease in the energy is observed as more bonds between particles can persist at lower temperatures. The two‐component system (Figure 2d) has a slightly higher average energy at higher $T^{*}$, compared to the one‐component system (Figure 2a), due to the reduced bonding availability imposed by the interaction specificity. Figure 2b,e shows the radial distribution functions, g(r), for the one‐component and two‐component systems, respectively, at select temperatures. The peaks in g(r) become sharper and persist to larger r values as the temperature is lowered, indicating an increase in positional order in both systems. In the one‐component system, this positional order is shorter ranged – the peaks flatten out more quickly as r increases. Figure 2c,f, where the two‐dimensional structure factors (S(k)) are shown for the one‐component and two‐component systems respectively, reveal a clear difference in the positional order between the self‐assembled structures. Even at low temperatures, the one‐component system exhibits a diffuse ring in the S(k) plot, indicating a lack of well‐defined hexagonal order in this system. In contrast, the S(k) plot for the two‐component system shows 6 isolated spots of hexagonal symmetry, indicating a high degree of hexagonal order. The lower‐intensity “satellite” peaks suggest that a polycrystalline structure is self‐assembled in the two‐component system.

**FIGURE 2:**
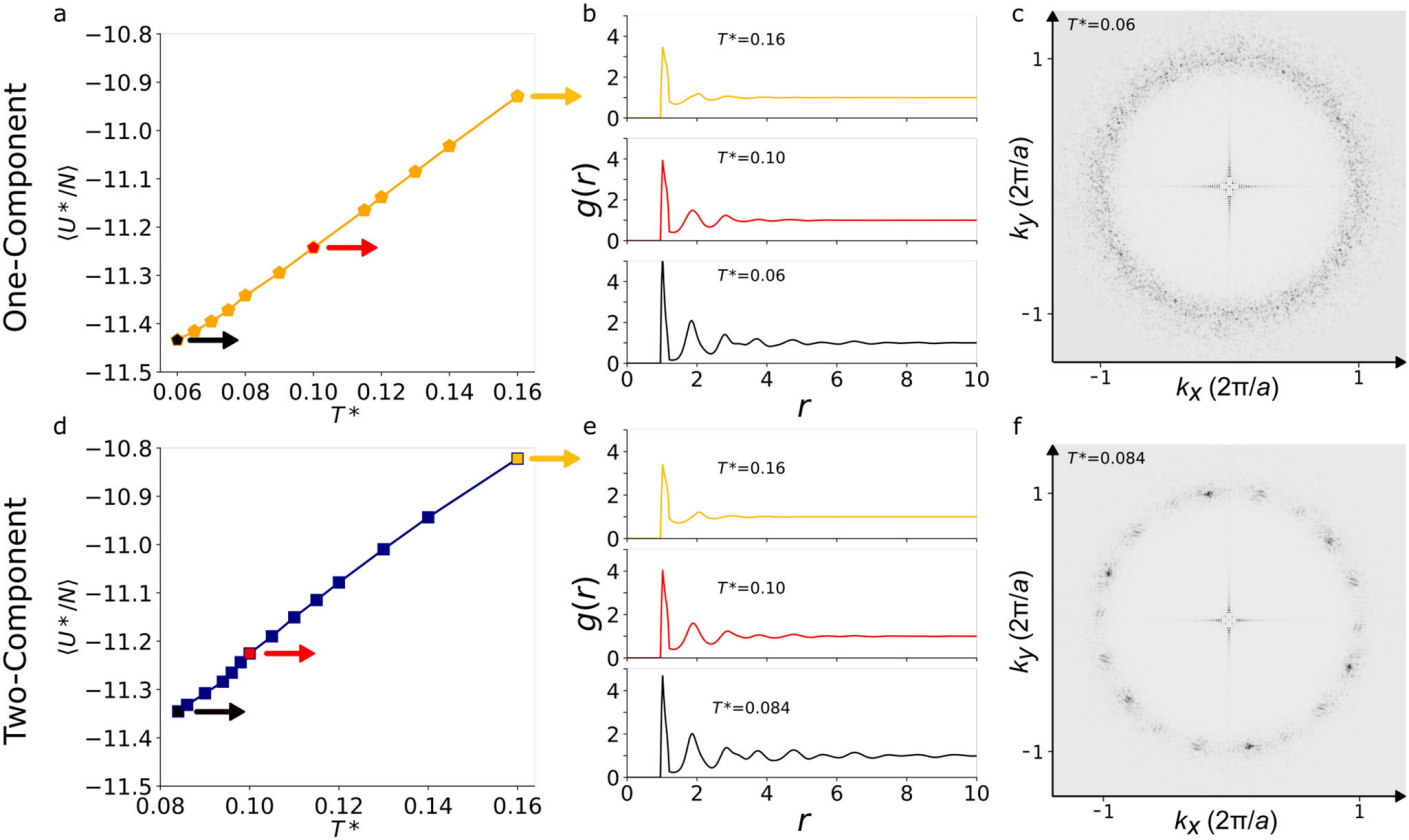
Self‐assembly of tetrahedral patchy particles on a two‐dimensional substrate. The results are for a one‐component system (a–c) and for a two‐component system (d–f), the latter with specific interactions between patches that allow for bonding only between different types of particles. Average energy per particle as the temperature is gradually reduced in the one‐component system (a) and in the two‐component system (d). (b,e) Radial distribution functions at representative temperatures, marked in (a) and (d), for the one‐component system (b) and for the two‐component system (e). The signatures of longer‐ranged positional correlations in the two‐component system are apparent at low temperatures. (c,f) Structure factor, S(k), in 2D for the lowest simulated temperature investigated for the one‐component system (c) and the two‐component system (f). Sharply defined spots of hexagonal symmetry imply the emergence of hexagonal order in the two‐component system at the temperature studied.

In Figure 3, we probe in further detail the emergence of crystalline order in the two‐component system in comparison to the one component system. To this end, Figure 3a,d shows the evolution of the ring statistics with temperature. For the analyses presented in Figure 3, we apply both positional and orientational criteria to place an edge between a pair of vertices (equivalent to the criteria for bonding between a pair of particles without considering the specificity of the interactions) while identifying rings in the underlying network. Upon cooling, we find a distribution of ring sizes, including 5‐ and 7‐member rings, in the one‐component system, but a strong preference for the 6‐member rings in the two‐component system, where 5‐ and 7‐member rings are practically absent. However, visual inspection of the configurations for the two‐component system reveals the presence of somewhat irregular 5‐ and 7‐member polygons, which can be captured in the ring statistics (see Figures S1 and S2) if only the distance criterion is used to place an edge between a pair of vertices in the underlying network. Further details of the two procedures for identifying rings are provided in Methods and Supporting Information (see Figure S1). Although the two procedures yield disparate results regarding the presence of 5‐ and 7‐member rings, they produce similar results for 6‐member rings, as evident in Figure S2. We do, however, note that the number of 6‐member rings is consistently underestimated when both positional and orientation criteria are applied. This is because the simple criterion based on a pair separation only is less stringent than the criteria used in the analyses presented in Figure 3.

**FIGURE 3:**
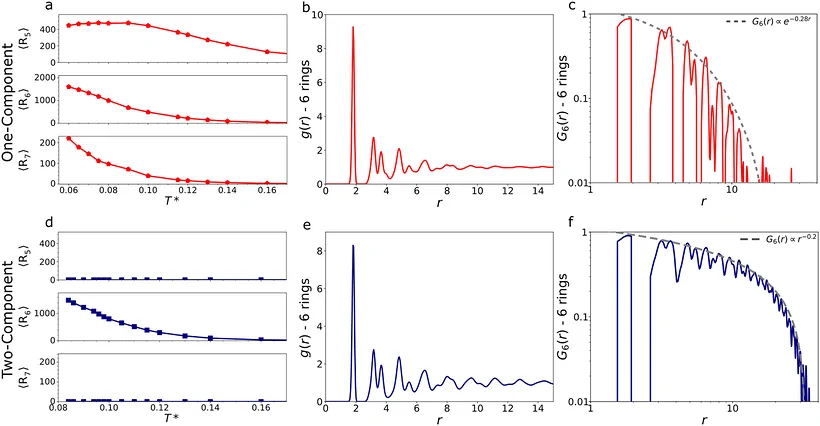
Promotion of the self‐assembly of a colloidal honeycomb crystal via suppression of topological defects. The results are for a one‐component system (a–c) and for a two‐component system (d–f), the latter with specificity of interactions to select for bonds between patches of two different types of particles exclusively. (a,d) Average number of rings, $R_{l}$ with size l (l∈[5 ‐ 7]), as a function of temperature for the (a) one‐component and (d) two‐component systems. The two‐component system shows no odd‐member rings. (b,e) Pair distribution functions for the centers of 6‐member rings in the (b) one‐component and (e) two‐component systems at the lowest temperature investigated. (c,f) Bond‐orientational correlation functions for 6‐member rings in the (c) one‐component system at $T^{*}=0.06$, (f) two‐component system at $T^{*}=0.084$. The one‐component system (c) shows a sharper decay than the two‐component system (f) as indicated by the fitted decay curves shown with dashed grey lines.

When we compare the pair distribution functions for the geometric centers of the 6‐member rings in the two systems, as shown in Figures 3b and 3e, a longer‐ranged correlation between the centers of the 6‐member rings in the two‐component system is evident, with persistent peaks to higher values of the separation. The 6‐member rings also show longer‐ranged orientational ordering in the two‐component case. The bond‐orientational correlation, $G_{6}\left(r\right)$, decays more rapidly for the 6‐member rings in the one‐component system than in the two‐component system (exponentially as opposed to algebraically, as shown in Figure 3c,f, respectively). The results presented in Figures 2 and 3 demonstrate the promotion of long‐range order in the course of self‐assembly in the two‐component system, compared to the one‐component system.

We now unravel the mechanism for the emergence of long‐range order in the two‐component system by analyzing common defect motifs, shown in Figure 4 where we apply the distance criterion alone to map the underlying network. Taking into account our findings in Figure S2, we argue that pentagonal and heptagonal structural motifs remain the prevalent defects to negotiate in the two‐component system as in the one‐component system, as evident in Figure 4a,b. Notably, with the distance only criterion, floating‐bond defects [37], where the particles have more than three neighbors, are identified in both systems. The histograms of nearest neighbor distances, $d_{i-j}$, shown in Figure 4c, reveal the spatial distributions of rings of certain sizes relative to each other. Here, $d_{i-j}$ stands for the distance to the center of the nearest i‐member ring from the center of a j‐member ring. A more pronounced peak at a small distance and a more rapidly decaying distribution with increasing distance for $d_{5-6}$ and $d_{7-6}$ in the one‐component system compared to the two‐component system indicate that in the former, the pentagonal and heptagonal defect motifs are more interspersed with the hexagonal structural motifs. This spatial distribution of defect rings disrupts the emergence of long‐range order in the one‐component case. The $d_{7-5}$ distributions in Figure 4c suggest that the dislocations (see Figure S3), also seen in a related experimental study [29], are more abundant in the one‐component system.

**FIGURE 4:**
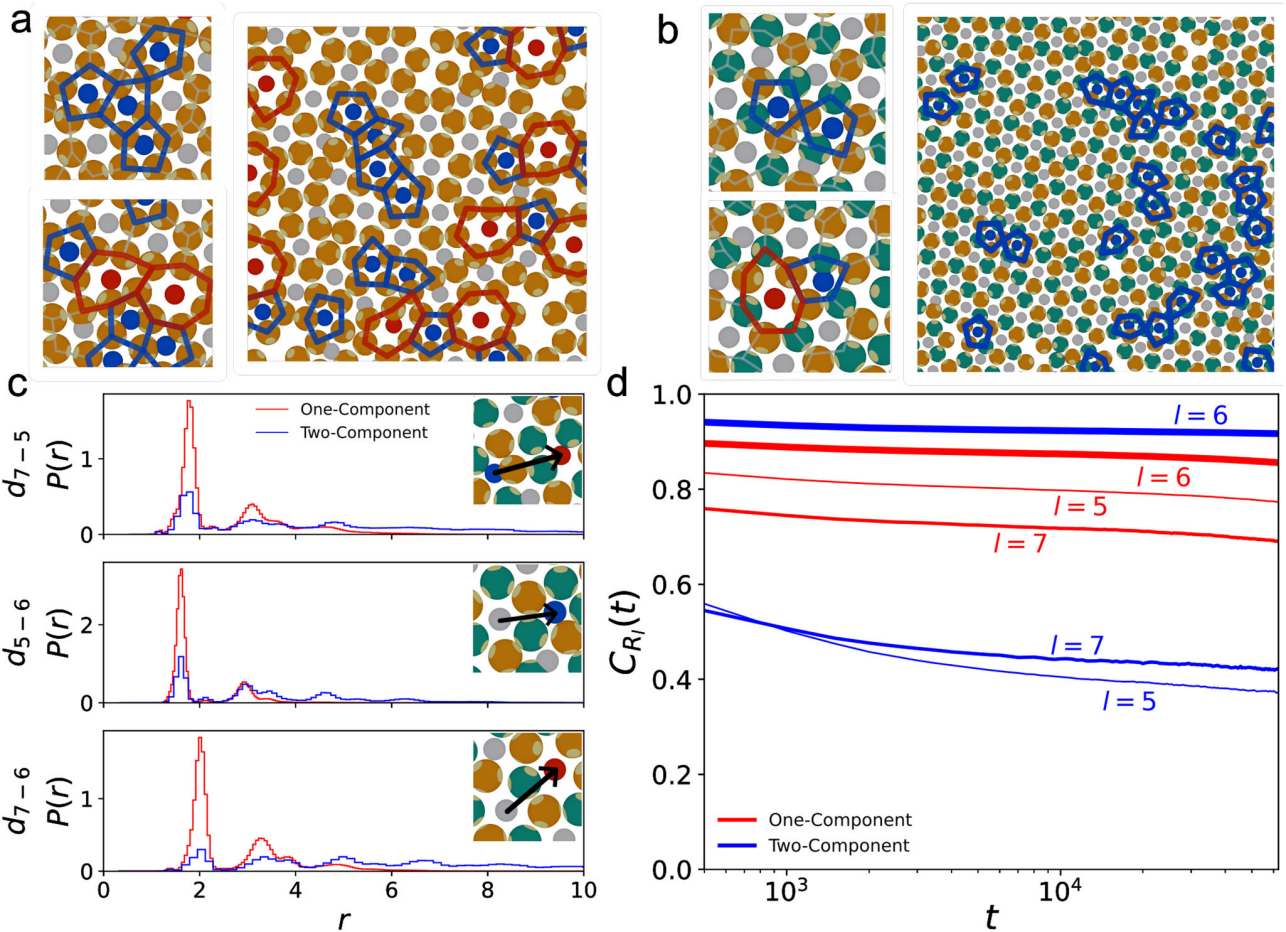
Spatial distributions of certain common defect motifs and their persistence. Certain common defect motifs are shown for the (a) one‐component system and (b) two‐component system. Here bonded neighbors are identified based only on the pair separation in the process of identifying rings. (c) Histograms of nearest neighbor distances, $d_{i-j}$, showing the spatial distributions of rings of certain sizes relative to each other. Here, $d_{i-j}$ stands for the distance to the center of the nearest i‐member ring from the center of a j‐member ring; note that $d_{i-j}$ and $d_{j-i}$ are not equivalent. Inset schematics show that for a given ring of size j, only the distance to the nearest ring of size i is recorded for the counting of the $d_{i-j}$ histograms. The suppression of topological defects in the two‐component system is evident in the top plot. (d) Ring autocorrelation functions for various ring sizes identified in both systems using only distance criteria. The plots represent the fraction of rings present in the system following t Monte Carlo cycles (i.e., we are taking a single Monte Carlo cycle to be the unit of time), independent of any ring opening events that may have occurred between the two time instants.

In contrast, the two‐component system typically displays pentagonal and heptagonal defect motifs at the edges of grains comprising the ideal hexagonal motifs (see Figure 4b and Figure S4). The specific interactions between the patches on different types of particles energetically destabilizes these odd‐member rings in the two‐component system compared to those in the one‐component system, thus allowing for annealing of common defect motifs in the two‐component system upon cooling. This energetic destabilization results in less persistent 5‐ and 7‐member rings in the two‐component system compared to the one‐component system, as evident in Figure 4d. Additionally, the 6‐member rings are more persistent in the two‐component system, presumably due to a cooperative effect that comes into play as the grain size comprising ideal hexagonal motifs grows in the two‐component system. We note that to enable fair comparison of the ring persistence the analysis in Figure 4 is carried out for both systems using configurations from temperatures with the same average energy per particle. This corresponds to $T^{*}=0.08$ for the one‐component system, and $T^{*}=0.084$ for the two‐component system, see Figure 2a.

To supplement the results presented above, the effects of density and patch size on the self‐assembly of the honeycomb crystal are investigated, as described in the Supporting Information. Although self‐assembly of the honeycomb crystal is observed for a range of densities after a one‐step cooling, especially in the two‐component system, the quality of the assembled crystal – assessed in terms of the percentage of 6‐member rings identified – appears to be better for higher particle number densities (see Figure S5). As a trend, we find that the number of 6‐rings present in a system – when subjected to a one‐step cooling from the fluid phase to a moderately low temperature – increases as the patch size of the tetrahedral patchy particles is increased (corresponding to a variation of the half‐opening angle from $13^{\circ }$ to $22^{\circ }$, see Figure S6). The robustness of our design to promote long‐range order in the course of the self‐assembly of a honeycomb crystal is thus well established.

The morphology of the structure self‐assembled from patchy particles with tetrahedrally oriented patches depends on the patch size. It has been shown that the patch half‐angle impacts the thermodynamic driving force of crystallization of tetrahedral patchy particles modeled by the Kern–Frenkel potential [33]. Other studies have shown that wider patches lead to arrested and glassy states [38, 39]. We note that the tetrahedral patchy particles considered here possess wider patches, relative to what would have been required had there been a trigonal arrangement of patches, which might be considered ideal for the self‐assembly of honeycomb structures. The reduced available bonding volume in a plane defined by the nearest three neighbors of a particle in a honeycomb structure necessitates an increased patch size in the present case with tetrahedrally arranged patches on the substrate.

### 2.2 Valley‐Hall Photonics

Having demonstrated that our two‐component assembly approach promotes the emergence of long‐range order we now turn to the photonic properties of the crystal. In order to generate a topologically nontrivial phase based on a honeycomb lattice either time‐reversal or spatial inversion symmetry must be broken [40, 41]. Time‐reversal symmetry can usually be broken by the application of a magnetic field [42, 43, 44], whilst spatial inversion symmetry can be broken by using two different “particles” in the honeycomb unit cell. This distinction is most commonly achieved in 2D structures using cylinders of differing radius and / or optical properties [45, 46, 47, 48, 49].

We start with a choice of two different dielectric constants for the two types of colloidal particles in our two‐component approach, see Figure 5a. This distinction of the two different particles allows for the breaking of spatial inversion symmetry, and opens a frequency gap between the first and second bands at the high symmetry **K** and $K^{\prime }$ points in the Brillouin zone (BZ). Figure 5a schematically shows the setup for our photonics calculations for both the one‐component and two‐component honeycomb crystals in a capped slab structure. The substrate layer, upon which the colloidal particles are self‐assembled in the present study, is considered in our photonics calculations as a dielectric slab. We also introduce a capping ‘superstrate’ layer to the top of the self‐assembled honeycomb crystal, introducing a mirror plane of symmetry through the slab. With these $\varepsilon_{r}=4$ sandwiching dielectric layers (also see Figure S7 for a schematic), we can separately consider TE (transverse electric) and TM (transverse magnetic) polarised light [50], and situate the first and second bands below the light cone, thereby confining the modes of interest to within the slab structure.

**FIGURE 5:**
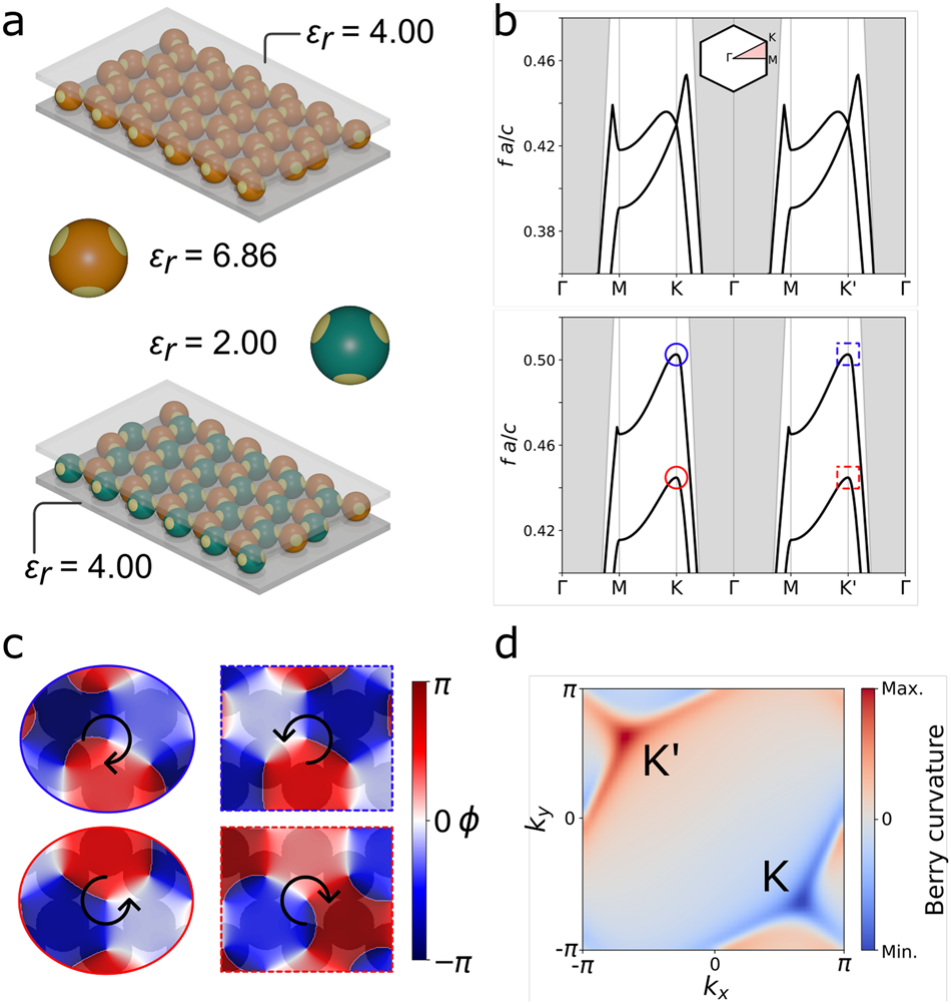
Colloidal valley photonic crystal achieved by dielectric contrast between particles on two sub‐lattices of a honeycomb lattice with broken inversion symmetry. (a) One‐component (top) and two‐component (bottom) honeycomb crystal slabs sandwiched between dielectric layers with dielectric constant $\varepsilon_{r}=4.00$. The orange and teal particles have dielectric constants of $\varepsilon_{r,A}=6.86$ and $\varepsilon_{r,B}=2.00$, respectively. (b) Honeycomb crystal slab photonic band structures. Top: Photonic band structures for the one‐component slab structure, showing degeneracy at the **K** and $K^{\prime }$ points. The 2D Brillouin zone of the honeycomb lattice is shown as an inset. Bottom: Photonic band structure for the two‐component system. Here, the broken inversion symmetry of the honeycomb structure is exploited by particles with sufficiently different dielectric constants and the degeneracy at the **K** and $K^{\prime }$ points is lifted. The **K** ($K^{\prime }$) point at band 1 and band 2 are highlighted with red and blue circles (squares) respectively. (c) Phase profiles of the electric field at the points highlighted in (b). We see opposite pseudo‐spin states for band 1 (lower) and band 2 (upper) at the **K** point (left). The same is true for band 1 and 2 at the $K^{\prime }$ point (right). (d) Colourmap showing the Berry curvature with local extrema at the **K** and $K^{\prime }$ points of the Brillouin zone.

In Figure 5b we show the TM band structures (computed by scanning wavevectors inside the 2D hexagonal BZ, see Figure 5b – inset) for light propagating in the plane bisecting the slab structure. The top panel of Figure 5b shows the first two TM bands for a one‐component colloidal honeycomb crystal of particles made from ${\mathrm{TiO}}_{2}$ (with dielectric constant $\varepsilon_{r}=6.86$), a popular material for photonics applications due to its low absorbance at optical wavelengths [51]. The crossing of the photonic bands at the **K** and $K^{\prime }$ points is evident, showing the same band topology as the Dirac points in the electronic band structure of atomic graphene [52, 53], and is a consequence of the symmetry of the honeycomb structure. The photonic band structure for the slab containing a two‐component honeycomb structure, with the dielectric constants of the two types of particles made sufficiently different ($\varepsilon_{r}=6.86$ and $\varepsilon_{r}=2.00$ in this case) is shown in the bottom panel of Figure 5b. The degeneracy at the **K** and $K^{\prime }$ points is lifted and a frequency gap is opened between the bands at these points. The values of $\varepsilon_{r}=6.86$ and 2.00 provide a sufficient contrast while still being experimentally attainable using ${\mathrm{TiO}}_{2}$ and common polymers such as PTFE. The phase profiles of the electric field at the **K** and $K^{\prime }$ points for these TM bands in Figure 5c show opposite behaviour. We see an anticlockwise phase progression at the **K** point for band 1 and a clockwise progression for band 2, while the reverse is true at the $K^{\prime }$ point.

To quantify this topological non‐triviality, we compute the Berry curvature, a measure of where phase change is occurring in the BZ [54]. In valley photonic topological insulators, the phase change is localized at the valleys – the reciprocal space neighborhoods of the **K** and $K^{\prime }$ points – allowing the symmetry here to be utilized for wave transport. Again, we restrict our calculations to the 2D hexagonal BZ and compute the Berry curvature for the two‐component slab structure, evaluating the modes at the z=0 plane bisecting the spheres in the capped slab structure. We compute contributions to the Berry phase as the discretized BZ is sampled by solving Maxwell's equations at each point [55, 56]; see Methods for details. Figure 5d shows how the Berry curvature varies over the BZ. As expected, we see localization of the Berry curvature of opposite signs in the valleys around the **K** and $K^{\prime }$ points.

Finally, we use the emergence of these opposite pseudo‐spin states for waveguiding applications. The different pseudo‐spin states at the **K** and $K^{\prime }$ points indicates that circularly polarized light will couple to these states depending on its handedness. We see from the phase profiles in Figure 5b that the handedness of the states is reversed between the **K** and $K^{\prime }$ points. We therefore construct a waveguide made from this structure to achieve unidirectional propagation of circularly polarized light, see Figure 6. The waveguide is constructed using two two‐component honeycomb slabs with inverse symmetry [46, 48, 57]. In the bottom slab, the high‐$\varepsilon_{r}$ particles occupy site A in the unit cell, and the low‐$\varepsilon_{r}$ particles occupy site B. This geometry is reversed in the top slab, resulting in two complementary structures, related to one another by a swapping of the two distinct sub‐lattices of the two‐component honeycomb lattice. The slabs are laterally shifted with respect to one another to break the mirror symmetry along the waveguide gap (see Figure 6a) [58] but remain coplanar. We note that an experimental realization of this waveguide geometry would require post‐processing of the self‐assembled honeycomb crystal structures to engineer the interface between the two slabs. The inversion of particle positions is also reflected in the Berry curvature and thus pseudo‐spin states at the **K** and $K^{\prime }$ points. In the top half of the waveguide slab, the **K** point shows an anticlockwise phase progression for band 1 while the bottom half shows a clockwise progression at the **K** point for band 1; the reverse is true for the $K^{\prime }$ point [59, 60]. This effect is demonstrated by the transmittance spectra shown in Figure 6b,c. In Figure 6b the waveguide, source, and detector are set up as shown in Figure 6a, and we observe that right‐circularly polarized light (RCP) propagates to the right along the waveguide. In Figure 6c, the source and detector are flipped; we observe left‐circularly polarized light (LCP) traveling left down the waveguide from the source to the detector. The grey areas in both plots indicate frequencies outside the band gap. The transmittance plots are normalized by the spectrum for an empty slab structure with no honeycomb lattice; the dotted line at a transmittance value of 1 therefore corresponds to transmittance through this reference structure. Transmittance values below 1 then demonstrate propagation, which is inhibited for this handedness of circularly polarized light.

**FIGURE 6:**
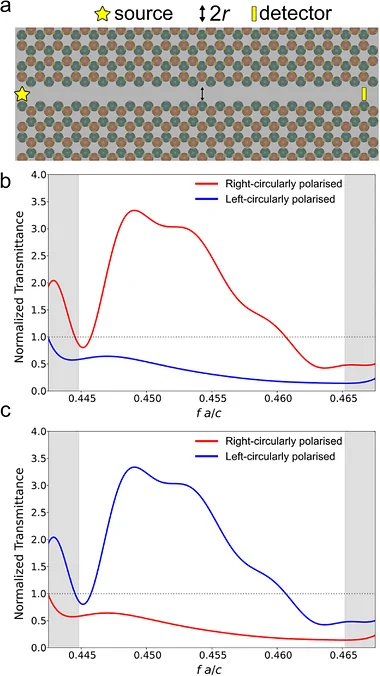
Unidirectional propagation of circularly polarized light through a valley photonic waveguide. (a) Top‐down schematic of the waveguide structure. The chiral light source and detector are placed on opposite sides of the waveguide, consisting of two complementary structures. The two structures are related by a swapping of the distinct sub‐lattices of the honeycomb structure. The separation between complementary structures is set to 2r, r being the particle radius. (b) Transmittance spectra for right‐ and left‐circularly polarized light (RCP and LCP, respectively) through the waveguide when the source and detector are placed in the positions shown in (a). (c) Transmittance spectra for RCP and LCP through the waveguide when the source and detector swap their positions. Transmittance plots are normalized with respect to an empty slab structure; two dielectric layers with no honeycomb slab between them. Along the waveguide, RCP propagates to the right and LCP to the left.

A similar result is obtained for a Z‐shaped waveguide (see Figure S8). We find that the preferential unidirectional propagation is retained to a reasonable extent even when self‐assembled crystallites with defects (Figure S9), a range of particle size polydispersity values (Figure S10), and deviations from ideal lattice positions (Figure S11) are considered. Furthermore, this behaviour persists for waveguide structures with both polydispersity in particle size and deviations from the ideal lattice positions to representative values (see Figure S12). We note that with increasing positional disorder, the floppy modes present in the crystal become soft and somewhat spatially localized [61]. The results presented here are shown in reduced units, reflecting the scalability of Maxwell's equations; colloidal particles with diameters in the range of ∼100−200 nm will bring the operational frequencies into the visible range. Rutile is widely used in colloidal materials for visible‐light photonics, especially because of its high refractive index, combined with its chemical and thermal stability. Although rutile remains fairly transparent across much of the visible spectrum, its dispersion becomes strong towards the near‐UV [62]. It should be noted that strong frequency dispersion in the short‐wavelength visible range can substantially affect the stability and robustness of the topological state, increasing its sensitivity to disorder.

## 3 Discussions

The specificity of particle interactions in the two‐component system is critical to the success of the programmed self‐assembly of the honeycomb structure, according to our design rules. This specific binding between the two particle species can be achieved using a complementary DNA coating [7, 63, 64, 65, 66, 67]. Such complementary DNA coating on patches has been realized [65], using patches functionalized with amidine. The amidine functionalization enables site‐specific coating of DNA with single‐stranded sticky ends via a biotin‐streptavidin‐biotin linkage, where biotin molecules are conjugated to the amidine patch. The binding between the patches of particles A and B is then realized by hybridizing DNA oligomers on the different patches. The oligomers used are complementary DNA strands designed to bind selectively to each other. The strength of DNA‐mediated interactions can be controlled by the length of DNA sticky ends and the grafting density. The hybridization of the complementary strands is fully reversible with temperature, allowing particle assembly to be controlled by varying temperature.

To further support experimental realization, we test whether the success of the programmed self‐assembly of the honeycomb structure can tolerate deviations from the perfect interaction specificity (i.e., from the scenario where $\varepsilon_{\mathrm{AA}}$ = $\varepsilon_{\mathrm{BB}}$ = 0). We perform additional Monte Carlo simulations for systems of N=960 tetrahedral patchy particles on a substrate subject to gradual cooling, in which we vary $\varepsilon_{\mathrm{AA}}$ and $\varepsilon_{\mathrm{BB}}$ relative to $\varepsilon_{\mathrm{AB}}=1$, while keeping $\varepsilon_{\mathrm{AA}}=\varepsilon_{\mathrm{BB}}$. We find that indeed the ring‐selection rules implemented are robust over a wide range of values for $\varepsilon_{\mathrm{AA}}/\varepsilon_{\mathrm{AB}}$ (where $\varepsilon_{\mathrm{AA}}/\varepsilon_{\mathrm{AB}}=0$ corresponds to the perfect interaction specificity scenario), as shown in Figure S13. It is evident that the ring‐selection rules do not begin to fail until $\varepsilon_{\mathrm{AA}}/\varepsilon_{\mathrm{AB}}>0.7$.

## 4 Conclusion

Colloidal self‐assembly offers a scalable and sustainable approach to the fabrication of complex structures for photonics and beyond. However, one of the challenges faced by this approach lies in the practically unavoidable formation of defects, which tend to compromise the photonic properties of self‐assembled colloidal materials. Structures endowed with topological properties are robust to such disorder or imperfections and therefore make ideal targets for programming colloidal self‐assembly. Here, we demonstrate design rules for a two‐component system of tetrahedral patchy particles on a substrate to self‐assemble into a honeycomb structure, while simultaneously suppressing the formation of topological defects in real space and imparting topological photonic properties in reciprocal space. This work thus paves the way for the scalable fabrication of a robust topological valley photonic crystal operating at optical frequencies via the self‐assembly of colloidal patchy nanoparticles.

The present work opens several avenues for further research. An exciting line of inquiry for gaining a fundamental understanding of the interactions between real‐space and reciprocal‐space topological phenomena is to explore whether residual topological defects in the two‐component colloidal honeycomb structure give rise to robust topological bound states [68, 69]. Another pertinent question is how the melting of the open crystal in the two‐component system compares and contrasts with that in the one‐component system in connection with the scenarios predicted by the celebrated Kosterlitz–Thouless–Halperin–Nelson–Young (KTHNY) theory [70, 71, 72, 73, 74] (see Figure S14 for preliminary results). Given that these scenarios have been largely investigated in simulations and experiments with closed‐packed 2D colloidal systems [75, 76], such a line of inquiry should be of interest, but is beyond the scope of the present study. Finally, it should be of interest to explore whether the strategy we present here is transferable to design diamond‐structured colloidal crystals for the investigation of valley contrasting physics in 3D, which is an emerging line of inquiry [77, 78].

## 5 Methods

### 5.1 Experimental assembly studies

Experimental studies of colloidal assembly are performed on patchy particles, made from polystyrene (PS), exhibiting tetrahedrally coordinated patches of 3‐(trimethoxysilyl)propyl methacrylate (TPM) [79]. The patchy particles attract each other in binary solvents due to critical Casimir forces [80, 81, 82]. The particles have a diameter of d=2.0μm and a patch diameter $d_{p}∼0.2\;\mu m$, allowing only single patches to bind with each other. The magnitude of the patch‐patch attraction is controlled by the correlation length of the binary solvent, set by the temperature offset $\Delta T=T_{c}-T$ from the solvent critical temperature, $T_{c}$ [80, 83]. The colloidal suspension is injected into a glass capillary with hydrophobically treated walls, to which the particles become adsorbed via one of their patches and thus become pseudo‐trivalent. To observe near‐equilibrium assembly, we slowly approach $T_{c}$ in small temperature steps of $0.05^{\circ }C$ starting from $\Delta T=0.25^{\circ }C$, leaving the sample to equilibrate for four hours at each step. This protocol results in a gradual increase of the strength of the patch‐patch attraction, ensuring a near‐equilibrium route to particle assembly. We image the assembled structure using confocal laser scanning microscopy. In the microscope images, the fluorescently labeled patches appear bright on a dark background, allowing determination of the particles' orientation and bond angles with their neighbors. The particle‐level imaging reveals that the pseudo‐trivalent particles diffuse freely along the surface and assemble into honeycomb crystallites with frequent defects, predominantly five‐ and seven‐member rings.

### 5.2 Model

The patchy particles investigated in the present study are hard spheres, decorated with four attractive circular patches on their surface in a tetrahedral arrangement. Three of the patches are available for ‘bonding’ to other particles, whilst the fourth anchors the particle to the surface. The interactions between patches are described by the Kern–Frenkel potential [36]:

(1) $$v_{ij}\left(r_{ij},\Omega_{i},\Omega_{j}\right)=v_{ij}^{\text{HS}}\left(r_{ij}\right)+v_{ij}^{\text{SW}}\left(r_{ij}\right)\sum_{\alpha ,\beta =1}^{4}f\left(r_{ij},{\hat{n}}_{i}^{\alpha },{\hat{n}}_{j}^{\beta }\right)$$

with $r_{ij}=\left|r_{ij}\right|$ the center–center distance between particles i and j, $\Omega_{i}$ and $\Omega_{j}$ describe the orientations of particles i and j, respectively, and $v_{ij}^{\text{HS}}$ is the hard‐sphere potential:

(2) $$v_{ij}^{\text{HS}}\left(r_{ij}\right)=\left\{\begin{array}{cc} \infty & \text{if}\;r_{ij}<\sigma ,\\ 0 & \text{otherwise} \end{array}\right.$$

with σ being the hard‐sphere diameter. Here, $v_{ij}^{\text{SW}}$ is a square‐well potential, which is given by

(3) $$v_{ij}^{\text{SW}}=\left\{\begin{array}{cc} -\varepsilon & \text{if}\;\sigma \leq r_{ij}\leq \left(1+\delta \right)\sigma ,\\ 0 & \text{otherwise} \end{array}\right.$$

where ε is the well depth and δ controls the range of attraction. The parameter δ is set to a value of 0.2 for all simulations performed in this study. The factor of $f(r_{ij},{\hat{n}}_{i}^{\alpha },{\hat{n}}_{j}^{\beta })$ controls the angular dependence of the interaction between patches:

(4) $$f\left(r_{ij},{\hat{n}}_{i}^{\alpha },{\hat{n}}_{j}^{\beta }\right)=\left\{\begin{array}{cc} 1 & \text{if}\;{\hat{n}}_{i}^{\alpha }\cdot {\hat{r}}_{ij}>\cos\;\theta \;\text{and}\;{\hat{n}}_{j}^{\beta }\cdot {\hat{r}}_{ji}>\cos\theta ,\\ 0 & \text{otherwise} \end{array}\right.$$

Here, ${\hat{n}}_{i}^{\alpha }$ is a normalized vector from the center of particle i to the centre of patch α on the particle surface. Similarly, ${\hat{n}}_{j}^{\beta }$ is a normalized vector from the center of particle j to the center of patch β on its surface. ${\hat{r}}_{ij}$ is the normalized center–center vector between particles i and j. θ is the patch half‐angle, controlling the width of a patch. In order to ensure that one patch interacts with only one other patch, we set $\theta =25^{\circ }$ for all patches. In Figure S6 we demonstrate the sensitivity of the honeycomb assembly to this patch angle, demonstrating that 6‐member rings are preferentially formed for θ values over $19^{\circ }$.

In the one‐component system, the particles are considered identical in size, as well as in terms of interaction range and strength. In the two‐component system, there are two types of particles, labeled A and B, such that the patches on A particles interact favorably only with the patches on B particles. This specificity of interactions is implemented by setting $\varepsilon_{\text{AA}}=\varepsilon_{\text{BB}}=0$ and $\varepsilon_{\text{AB}}=\varepsilon$, where the subscripts denote A‐A, B‐B and A‐B interactions, respectively.

#### 5.2.1 Surface Implementation

To model the experimental system where particles are confined to a surface [29, 84], we apply the same KF potential to the surface‐patch interaction. This interaction is described by the surface potential

$$v_{is}\left(r_{is},\Omega_{i}\right)=v_{is}^{\text{surf}}\cdot f\left(r_{is},{\hat{n}}_{i}^{\alpha }\right)$$

with $r_{is}=\left|r_{is}\right|$ the distance from the center of particle i to the surface. The surface interaction is defined by:

$$v_{is}^{\text{surf}}=\left\{\begin{array}{cc} -\varepsilon_{\text{surf}} & \text{if}\;r_{is}\leq \left(1+\delta \right)\frac{\sigma }{2}\\ 0 & \text{otherwise.} \end{array}\right.$$

The factor of $\frac{1}{2}$ here compared to the square well above ensures that we retain a monolayer of particles on the surface. As above, f controls the angular dependence of the patch‐surface interaction:

$$f\left(r_{is},{\hat{n}}_{i}^{\alpha }\right)\left\{\begin{array}{cc} 1 & \text{if}\;{\hat{n}}_{i}^{\alpha }\cdot {\hat{r}}_{is}>\cos\;\theta \\ 0 & \text{otherwise.} \end{array}\right.$$

${\hat{n}}_{i}^{\alpha }$ is a normalized vector from the centre of particle i to the center of patch α on the particle surface and ${\hat{r}}_{is}$ is the normalized vector between the center of particle i and the surface. We set the strength of patch‐surface interaction to be 10× stronger than that of the patch‐patch interaction, i.e. $\varepsilon_{\text{surf}}=10\varepsilon$. The particles are thus heavily biased to remain anchored near the substrate. Note that there is a finite range for the possible z‐coordinates of all particles in the system at the temperatures of interest. Patchy particles have been studied in the literature in a strictly 2D system, where the positions of the particles are confined to a plane and out‐of‐plane rotations are ignored [85]. The patch‐surface interaction range, together with the patch half‐angle, θ, is chosen to avoid binding of multiple patches to the surface. Longer‐range interactions and wider patches are more likely to result in particles anchoring to the substrate via multiple patches. Our approach to modeling thus yields a quasi‐2D system of pseudo‐trivalent particles that more closely mimics the experimental conditions [29].

### 5.3 Simulations of Self‐Assembly

We investigate the self‐assembly of two systems ‐ one a one‐component system of pseudo‐trivalent patchy particles and the other a two‐component system of 1:1 pseudo‐trivalent patchy particles with attractive interactions only between particles of different type. We employ the in‐house developed software package, PaSSion, to perform Monte Carlo simulations of the systems of patchy particles in the canonical ensemble. Particles are treated as rigid bodies and orientational degrees of freedom represented by quaternions. Trial moves consist of particle translations or rotations, chosen randomly with equal probability. Each system, consisting of N=5,800 patchy particles, is simulated using an orthorhombic box just large enough to accommodate a 58×50 grid of honeycomb unit cells for a particle number density of 0.72 across the simulation box surface. Both systems are subject to gradual cooling from an isotropic fluid bound to a surface spanning the *xy* plane of the simulation box with periodic boundary conditions. The system is then gradually cooled through successive simulations at progressively lower temperatures, with each simulation starting from the final configuration of the previous one. In each case, we ensure that the system equilibrates around a constant average energy per particle before further reducing the temperature.

### 5.4 Structure Factor

The structure factor is given by

(5) $$S\left(k\right)=\frac{1}{N}〈\sum_{i,j}^{N}e^{-ik(r_{i}-r_{j})}〉$$

where $r_{i}$ and $r_{j}$ denote the positions of particles i and j, respectively [86]. We plot the structure factor resolved in two dimensions as $S(k_{x},k_{y})$ with the |k| range restricted to values corresponding to nearest‐neighbor distances.

### 5.5 Bond‐Orientational Correlation Function

To quantify the six‐fold rotational symmetry, we use the bond‐orientational correlation function $G_{6}\left(r\right)$ [86]:

(6) $$G_{6}\left(r_{kl}\right)=\left\langle \psi_{6}^{*}\left(r_{k}\right)\cdot \psi_{6}\left(r_{l}\right)\right\rangle$$

$\psi_{6}\left(r_{k}\right)$ is the local hexatic bond‐orientational order parameter of particle k at position $r_{k}$ [87], given by

(7) $$\psi_{6}\left(r_{k}\right)=\frac{1}{n_{l}}\sum_{l=1}^{n_{l}}e^{i6\theta_{kl}}$$

Here, $n_{l}$ is the number of nearest neighbors, l, around particle k, and $\theta_{kl}$ the angle between the vector separating k and l and a fixed axis. For a 2D solid, the value of $G_{6}\left(r\right)$ reaches a constant (non‐zero) value at large r. For an isotropic 2D fluid phase, $G_{6}\left(r\right)$ decays exponentially.

### 5.6 Rings Analysis

We utilize a graph theory approach to identify and characterize the rings formed by particles in our simulations. Each particle is assigned as a vertex in a periodic, undirected graph representing the simulated system. We discuss above two methods for placing an edge between a pair of vertices to construct such graphs: in one method, both positional and orientational criteria for the particles represented by the vertices are used to place an edge if the particles are bonded in the description of the Kern–Frenkel potential; in the other method, the orientational criterion is ignored and an edge is drawn between these vertices if the particles are within a certain separation distance. See Figure S1 for further discussion of these criteria. In each case, the average number of rings of size l, $\left\langle R_{l}\right\rangle$, reported is for simple, chordless, closed loops of size l identified in the graph. For computational efficiency, we use the limit 3≤l≤9. Averages are taken over 1000 configurations following equilibration at every temperature.

### 5.7 Persistence of Rings

To obtain a quantitative estimate of the persistence of the rings formed by the patchy particles along the Monte Carlo trajectories, we calculate the ring autocorrelation function, $C_{R_{l}}$, defined as

(8) $$C_{R_{l}}\left(t\right)=〈\frac{\sum_{i=1}^{R_{l}\left(t^{\prime }\right)}\sum_{j=1}^{R_{l}\left(t^{\prime }+t\right)}H\left(O_{i},O_{j}\right)}{R_{l}\left(t^{\prime }\right)}〉$$

where $R_{l}\left(t^{\prime }\right)$ is the total number of rings of size l in the network at the instant of time, $t^{\prime }$, and $H(O_{i},O_{j})$ is a step function that takes on a value of unity if rings $O_{i}$ and $O_{j}$ are of the same size and contain the same particles, and is zero otherwise. We take the average over multiple time origins $t^{\prime }$. Similar to the bond autocorrelation function applied to hydrogen‐bond networks of water [88, 89], the ring autocorrelation function represents the fraction of rings present in the system following t Monte Carlo cycles (i.e., we are taking a single Monte Carlo cycle to be the unit of time), independent of any ring opening events that may have occurred during the lag between the two instants of time.

### 5.8 Photonic Calculations

We utilize the open source MIT Photonic Bands package (MPB) [90] to compute band structures and visualize the electric fields. MPB computes fully vectorial eigenmodes of Maxwell's equations with periodic boundary conditions by using conjugate‐gradient minimization of the block Rayleigh quotient in a plane wave basis.

The capped slab structures are modeled using a rhombic prism unit cell with lattice vectors $a=\sqrt{3}\sigma \left(\frac{\sqrt{3}}{2}\hat{x},\frac{1}{2}\hat{y}\right)$, $b=\sqrt{3}\sigma \left(\frac{\sqrt{3}}{2}\hat{x},-\frac{1}{2}\hat{y}\right)$ and $c=\left(6.4|a|+\sigma \right)\left(\hat{z}\right)$, the dielectric colloidal particles are just‐touching spheres with diameter σ=1. For calculations of the one‐component band structure, the spheres have dielectric constant $\varepsilon_{r}=6.86$, while for the two‐component system, the dielectric constants are $\varepsilon_{r}^{A}=6.86$ and $\varepsilon_{r}^{B}=2.00$ for the two particles, comparable to those of rutile and silica. This dielectric contrast enables a sufficiently wide frequency gap to emerge at the **K** point for our two‐component system. With this dielectric contrast between the two sublattices, the dielectric constant for the slab is varied to situate the modes of interest below the light cone, such that they are confined to the slab structure. For all calculations, the spheres are situated in contact with two layers of dielectric with thickness 0.2|a| and dielectric constant $\varepsilon_{r}=4.00$. We compute band structures with a computational grid resolution of 32 pixels per lattice unit, and a mesh resolution of 16 for dielectric constant averaging at each grid point.

### 5.9 The Berry Curvature

To compute the Berry curvature we use a discretized Brillouin zone (BZ) consisting of a 24×24 grid of k‐points. Solving for the frequency and modes of each band, n, at each k‐point gives us the electric fields $E_{n}\left(k_{x},k_{y}\right)$ across the unit cell. The Berry curvature, $\gamma_{n}\left(k\right)$, is given by

$$\gamma_{n}\left(k\right)=\nabla_{k}\times A_{n}\left(k\right)$$

with $A_{n}$ the Berry connection:

$$A_{n}\left(k\right)=i\langle E_{n}\left(k\right)|\nabla_{k}\left|E_{n}\left(k\right)\right\rangle$$

In the discretized space of a 2D BZ, with small enough steps between k‐points, we can write the Berry curvature in a discrete cell within the BZ as: [55, 56]

$$\gamma_{n}\left(k_{x},k_{y}\right)=-\text{Im}\ln\left(\left\langle E_{n,1}|E_{n,2}\right\rangle \left\langle E_{n,2}|E_{n,3}\right\rangle \left\langle E_{n,3}|E_{n,4}\right\rangle \left\langle E_{n,4}|E_{n,1}\right\rangle \right)$$

where Im denotes taking the imaginary part, and

$$\left\langle E_{n,a}|E_{n,b}\right\rangle =\sum_{p,q}E_{n,a}^{*}\left(p,q\right)\varepsilon \left(p,q\right)E_{n,b}\Delta s$$

a,b={1,2,3,4} denote four k‐points which define the vertices of a cell in the discretized BZ. The p,q values denote the ${\left(p,q\right)}^{\text{th}}$ discretized cell with area Δs in the plane bisecting the slab structure.

### 5.10 Transmittance Spectra

Transmittance spectra for circularly polarized light traveling along the waveguide are calculated using the open‐source finite‐difference time domain package MEEP [91]. Two finite slabs of the two‐component honeycomb structure make up the waveguide, each comprised of 20 unit cells in the x direction and 12 unit cells in the y direction. Dielectric slabs are situated above and below the honeycomb layers. The simulation cell is defined with a Yee grid of 16 points per unit cell and perfectly matched layers are positioned on all sides. A Gaussian pulse source is defined on one end of the waveguide, with energy flux measured on the other side. Circularly‐polarized sources are generated by overlapping two linearly‐polarized sources, which are $90^{\circ }$ out of phase with each other. The transmittance spectra are relative to those of an empty slab composed of two dielectric plates with no honeycomb structure.

## Author Contributions

D.C., P.S. and A.W. conceived the study; A.W. and A.N. performed the simulations; P.J.M.S. performed the experiments; A.W. and A.N. contributed to the development of software; all authors contributed to the analysis and/or interpretation of data; A.W. and D.C. wrote the manuscript with contributions from the others; all authors commented on the manuscript; D.C. supervised the project with contributions from A.D. and P.S.

## Conflicts of Interest

The authors declare no conflicts of interest.

## Supporting information




**Supporting File**: smll75515‐sup‐0001‐SuppMat.pdf.

## Data Availability

Numerical data supporting figures are available in the University of Birmingham eDATA Repository at https://doi.org/10.25500/edata.bham.00001782.
