# Supplementary material for: Self‐Assembly of a Colloidal Honeycomb Crystal in Silico for Topological Valley Photonics
Source: Small. 2026 Oct 4;22(62):e75515. doi: 10.1002/smll.75515 (PMC13742911; doi:10.1002/smll.75515)
Supplement: Supplementary file 1 [file SMLL-22-e75515-s001.pdf]

Self-Assembly of a Colloidal Honeycomb Crystal in Silico for Topological Valley Photonics

Adam Walker,[1,2] Andreas Neophytou,[2,3] Piet J. M. Swinkels,[4]
Angela Demetriadou,[5] Peter Schall,[4] and Dwaipayan Chakrabarti[2,6,a]

[1]*EPSRC Centre for Doctoral Training in Topological Design, University of Birmingham, Edgbaston, Birmingham B15 2TT, United Kingdom*
[2]*School of Chemistry, University of Birmingham, Edgbaston, Birmingham B15 2TT, United Kingdom*
[3]*Dipartimento di Fisica, Sapienza Università di Roma, Piazzale Aldo Moro 5, 00185, Roma, Italy*
[4]*Institute of Physics, University of Amsterdam, Amsterdam, The Netherlands*
[5]*School of Physics and Astronomy, University of Birmingham, Edgbaston, Birmingham B15 2TT, United Kingdom*
[6]*International Institute for Sustainability with Knotted Chiral Meta Matter (WPI-SKCM^2), Hiroshima University, 1-3-1 Kagamiyama, Higashi-Hiroshima, Hiroshima 739-8526, Japan*

Supporting Information

I. RING STATISTICS

In the main body of the article, we discuss two distinct methods for constructing a network to represent the systems of patchy particles considered in the present study. This network representation underpins the procedure we follow to identify rings, which are closed loops in a network. In both methods, the particles are represented as vertices or nodes. In the first method, we apply both positional and orientational criteria for bonding between two particles in the description of the Kern-Frenkel potential,[S1] and place an edge between two nodes when the corresponding particles are bonded in this description. An alternative is to ignore the orientations of particles and use a simple radial cutoff such that an edge between two nodes is placed whenever the corresponding particles are within a separation r_{ij}, which satisfies $\sigma \leq r_{ij} \leq 1.2\sigma$, σ being the particle diameter. In this second method, an edge is placed between two nodes, even if the corresponding particles do not have their patches aligned to form bonds. The two procedures for ring identification are illustrated in Fig. S1, where the underlying network is constructed by considering both positional and orientational criteria in Fig. S1(a) and only the positional criterion in Fig. S1(b). With a less stringent criterion in operation, more rings are identified in Fig. S1(b). The second method, which requires less information, could be more accessible to experimental techniques.

Ring statistics for 5-, 6-, and 7-member rings obtained in the two different identification methods are shown in Fig. S2a and S2d for the one-component and two-component systems, respectively. While the ring counts are different (more rings are identified with the distance-only criterion), the two-component system shows greater positional and orientational correlations (Figs. S2e-f) than the one-component system (Figs. S2b-c). It is important to note that the two different procedures followed to construct the underlying network in the process of identifying rings do not produce qualitatively different results for the self-assembled structures obtained for the one-component and two-component systems studied here.

II. DEFECTS

As noted in the main body of the article, we derive motivation for the present work from a recent experimental study,[S2] where the self-assembly of a honeycomb crystal from tetrahedral patchy particles interacting via critical Casimir forces on a substrate is investigated, highlighting the abundance of defects. Fig. S3 shows that the one-component system in the present study captures the common defect motifs observed in this experimental study, namely the 5- and 7-member rings as disclinations and the 5-7 pair as dislocation. This agreement can be seen as a validation of our simulation method.

We have noted that with the distance-only criterion, floating-bond defects,[S3] where the particles have more than three neighbours, are identified in both one-component and two-component systems. With the distance-only criterion,

[a] d.chakrabarti@bham.ac.uk

extended regions of 3- and 4-member rings, as marked in red in Fig. S4a, are predominantly observed in the two-component system, and appear to persist between crystalline grains, which feature long-range order. A zoomed-in view of one such region is shown in Fig. S4b, with the two grains on either side given black or grey backgrounds.

III. SELF-ASSEMBLY AT DIFFERENT DENSITIES

To supplement the self-assembly study at a given density presented in the main body of the manuscript, we investigate the effects of density on the self-assembly. To this end, a smaller system size is considered with $N = 960$ particles, and the system is subject to one-step cooling from an isotropic fluid phase at an initial temperature of $T^* = 0.20$ to several temperatures. The results of the self-assembly studies at different densities are summarised in Fig. S5. In order to quantify the degree of crystallinity in the self-assembled configurations, the percentage of 6-member rings identified using our orientational criteria is taken to be a measure of crystallinity present and is shown by a colour bar. The two-component system in Fig. S5b clearly shows a higher proportion of 6-member rings across the temperatures and densities studied than the one-component system in Fig. S5a. Furthermore, the quality of the assembled crystal appears to be better for higher particle number densities.

IV. EFFECTS OF PATCH SIZE

The patchy particles investigated in the self-assembly study presented in the main text have a fixed patch size, characterised by the half-opening angle of 25°. In order to investigate the effects of patch size on the self-assembly process, we perform further simulations, where systems of 960 tetrahedral patchy particles in a 1:1 two-component mixture at various particle number densities are subjected to one-step cooling from a fluid at $T^* = 0.20$. The systems differ in the half-opening angle characterising the patch size. The results of these simulations are summarised in Fig. S6, where the quality of the assembled crystal structure is once again assessed by the average number of 6-rings found using our orientation criteria. As the patch size of the tetrahedral patchy particles is increased, we find that the number of 6-rings present increases for one-step cooling to a temperatures $T^* = 0.08$. We again see that a higher particle number density results in a greater degree of crystallinity in the self-assembled structures.

Note that we report the average number of 6-member rings in Fig. S6, rather than reporting a percentage as in Fig. S5. This is due to the large variation in the number of rings identified in the patch angle study. For smaller patch sizes, there are very few rings, so small numbers of 6-member rings will yield large percentage values, making the systems appear more ordered than they are.

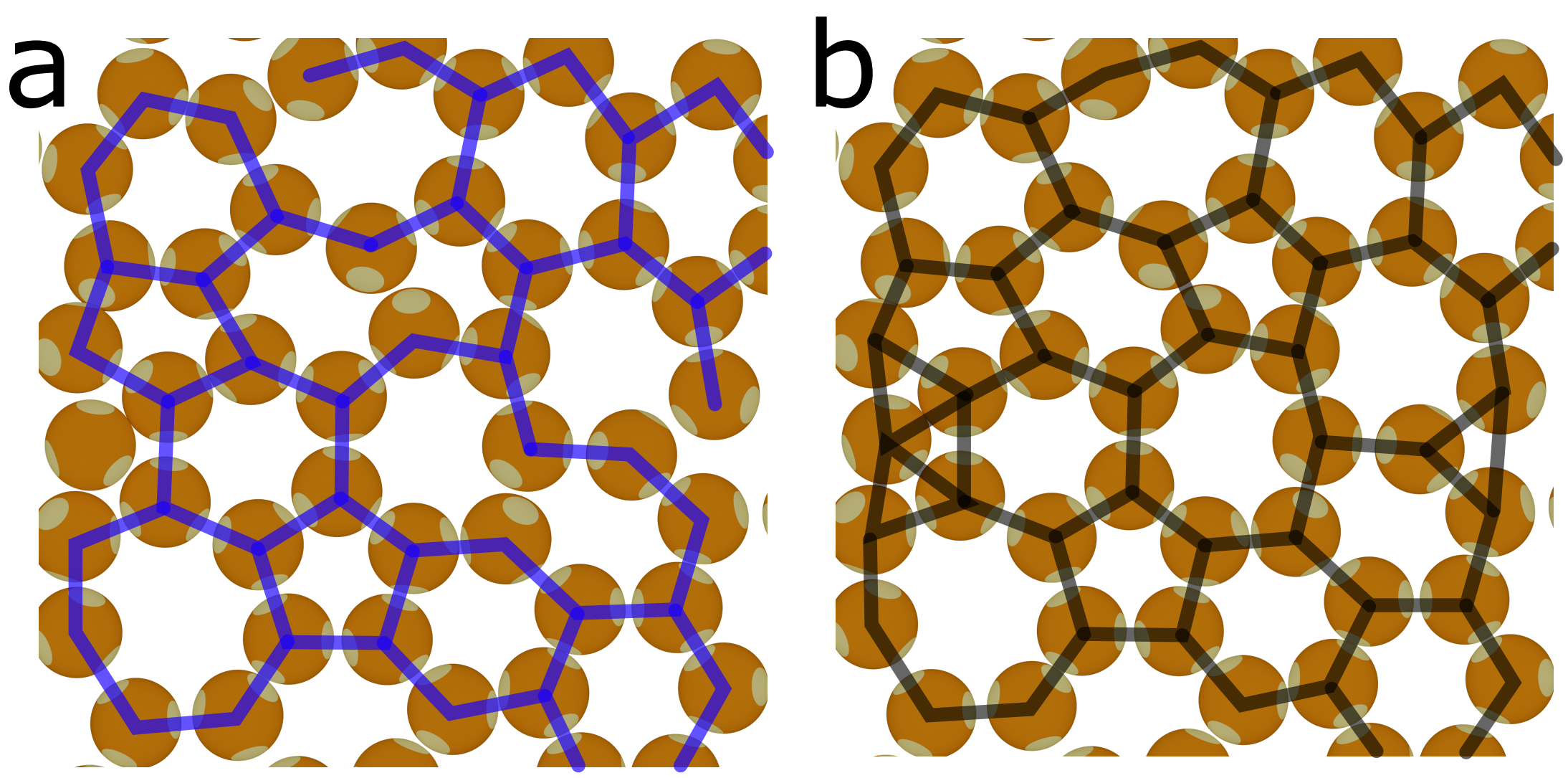

FIG. S1. Ring identification with and without consideration of particle orientation. Two distinct methods for constructing a network to represent the systems of patchy particles being considered in the present study are illustrated for identical configurations. An edge is placed between a pair of vertices (or nodes) representing the particles if (a) their positions and orientations satisfy the bonding criteria described in the Kern-Frenkel potential,[S1] (b) their positions are within a radial cutoff distance, irrespective of orientation.

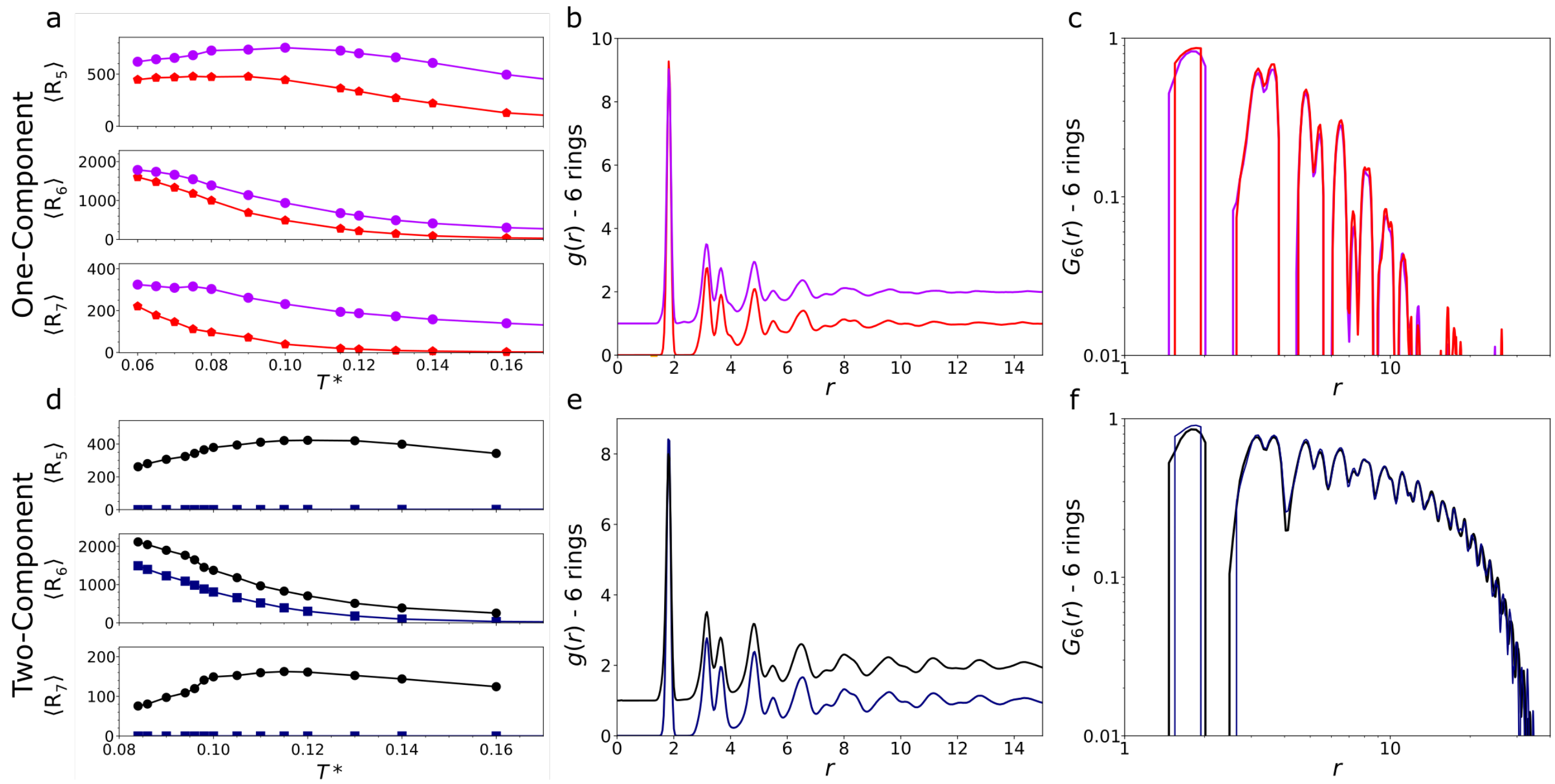

FIG. S2. Comparison of structural analyses using two different criteria for identifying rings. The results are shown for the one-component system (a-c) and the two-component system (d-f), using both the positional and orientational criteria or only the positional criterion for recognising neighbours in the process of identifying rings. Violet and black data points (or plots) correspond to the results obtained using the positional criterion only for the one- and two-component systems, respectively. Red and blue data points (or plots) are the results obtained when both the positional and orientational criteria are applied. (a, d) Average number of rings, R_l with size l ($l \in [5$ - $7]$), as a function of temperature for the (a) one-component and (d) two-component system. (b, e) Pair distribution functions for the centres of 6-member rings in the (b) one-component and (e) two-component systems at the lowest temperature investigated [$T^* = 0.06$ (one-component) and $T^* = 0.084$ (two-component)]. (c, f) Bond-orientational correlation functions for 6-member rings in the (c) one-component system at $T^* = 0.06$, (f) two-component system at $T^* = 0.084$. The one-component system (c) shows a sharper decay than the two-component system (f) for both analyses.

V. BAND STRUCTURE CALCULATIONS

We use a 3D unit cell to compute the band structures for the photonic crystal slabs presented in the main body of this manuscript. Fig. S7 presents a schematic of the unit cell used in the calculations. The periodic boundary conditions employed at the cell boundaries mean that no free-space modes are found in the calculations. Instead, we insert light cones to show that the bands of interest correspond to guided modes which are confined to the slab structure. Large vertical (z-axis) gaps between periodic images of the slab structure ensure that there is no coupling between adjacent layers.

VI. ROBUSTNESS OF THE WAVEGUIDE STRUCTURE

One key advantage of valley photonic topological insulators is their robustness to defects and backscattering. To probe the protection afforded by our system, we perform transmittance calculations using a Z-shaped waveguide; the results are presented in Fig. S8. We observe unidirectional propagation across this waveguide geometry with sharp corners.

We also conduct waveguide transmittance calculations using particle positions corresponding to the configurations sampled by Monte Carlo simulations in the present study. The results are presented in Fig. S9. Despite considering self-assembled crystallites with defects in these calculations, we still observe a strong unidirectionality effect along the waveguide geometry.

Since defects are practically unavoidable in crystal structures self-assembled from synthetic colloidal particles, which could have a degree of polydispersity in particle size and/or patch size, it is imperative that we investigate the impact

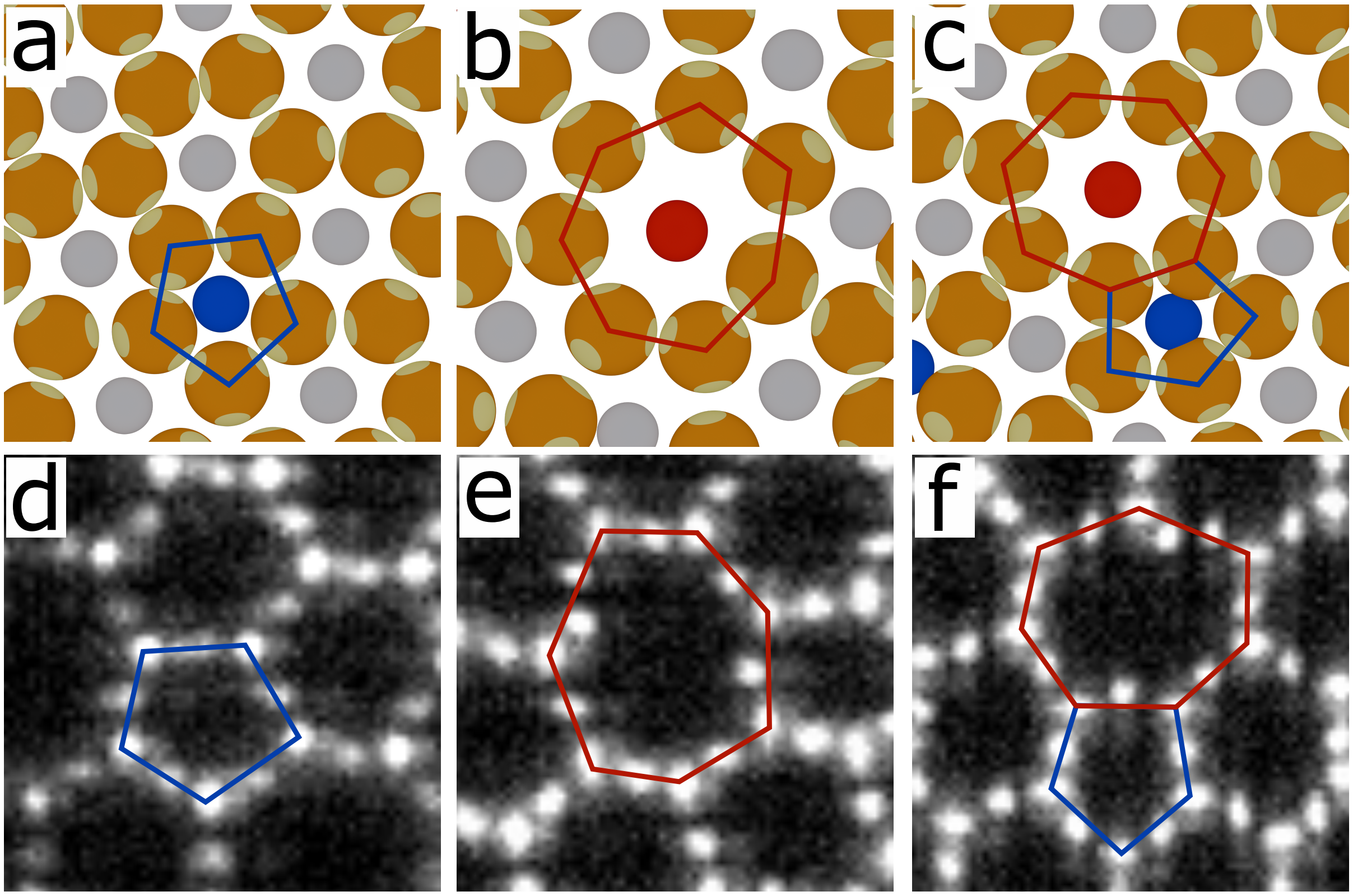

FIG. S3. Comparison with an experimental system. Common defect motifs identified in simulations of the one-component system (a-c) and in a recent experimental study[S2] (d-f).

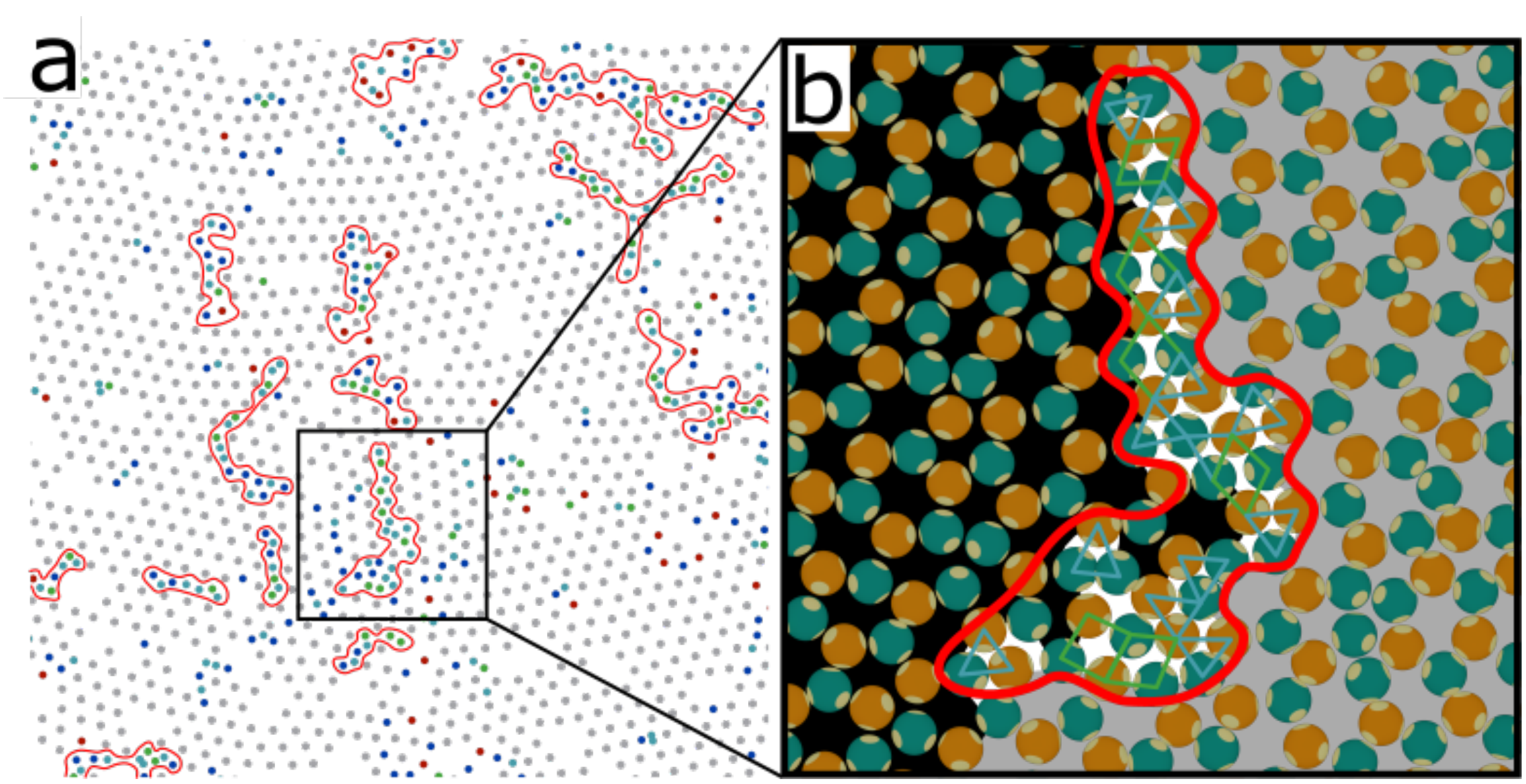

FIG. S4. Extended defect regions in the two-component system. Grain boundaries comprising 3- and 4-member rings. (a) Snapshot of the two-component system at $T^* = 0.084$, showing coloured circles to denote the geometric centres of rings identified using a radial cutoff, with 6-member rings in grey, 7-member rings in red, 5-member rings in blue, 4-member rings in cyan, and 3-member rings in green. Here, the particles are removed for clarity. Extended regions of 3- and 4-member rings are marked in red. (b) Zoomed-in view of one of these defect regions, again marked in red but now showing the particles. The two grains on either side of the defect region are distinguished with black or grey backgrounds.

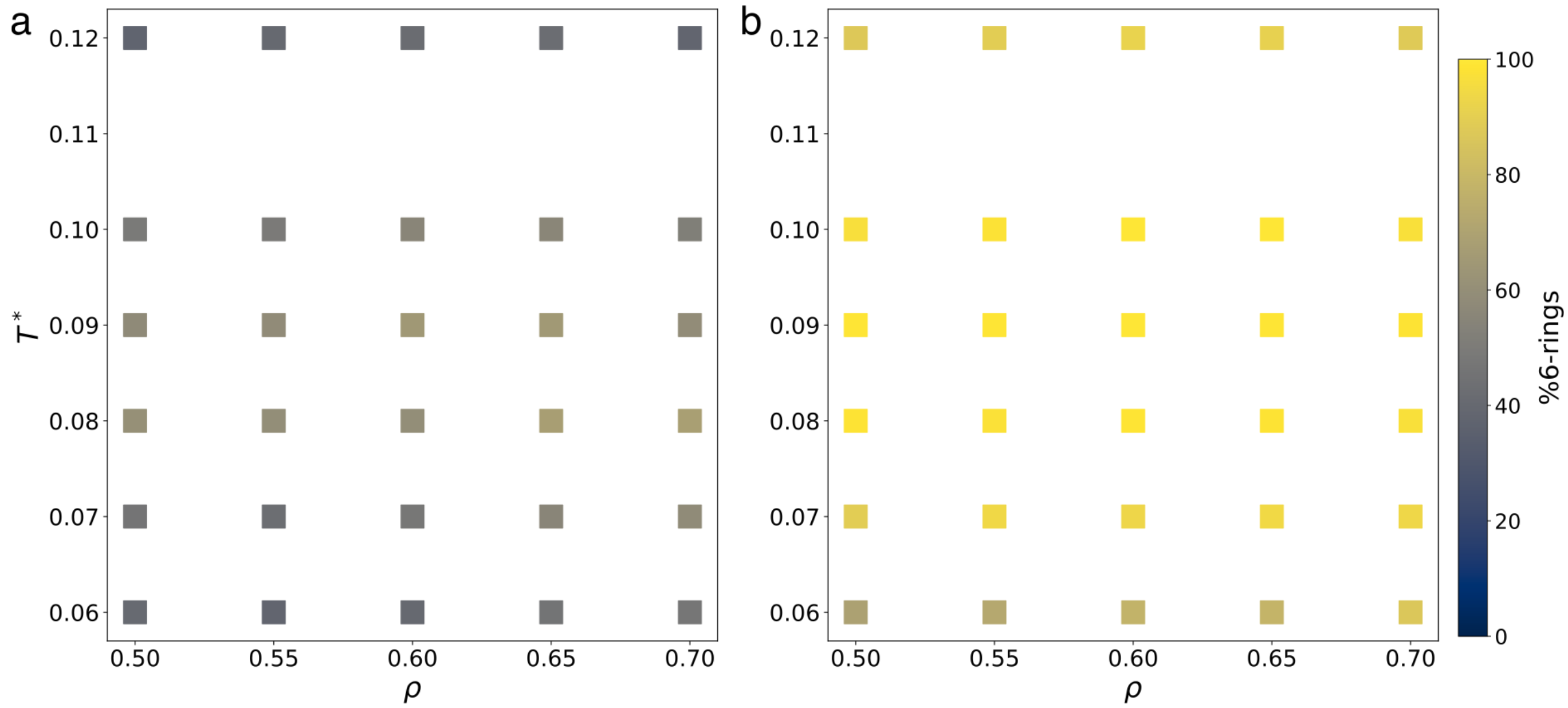

FIG. S5. Effects of the particle number density on the quality of the self-assembled crystal structures for both one-component and two-component systems considered. (a) and (b) present a summary of the results obtained for one-component and two-component systems, respectively, when a system is subject to a one-step cooling from a fluid at $T^* = 0.20$ to various temperatures T^* at different values for the particle number density $\rho^\star$. The colour-coding represents the quality of the self-assembled crystal structure measured in terms of the percentage of 6-member rings identified in the system according to our orientation-specific criteria.

of these deviations from the perfect crystal structure on the performance of the waveguide constructed.

A. Polydispersity

To assess the effects of the distribution in particle size on the waveguide performance, we incorporate polydispersity by drawing the particle radius, r, of each particle from a normal distribution centred at $r_0 = 0.5\sigma$ (for just-touching spheres) with a standard deviation of σ_r:

$$P(r) = \frac{1}{\sqrt{2\pi(\sigma_r)^2}} \exp\Big[-\frac{(r-r_0)^2}{2(\sigma_r)^2}\Big].$$

Transmittance spectra are computed for 10 separate geometries, with particle radii drawn from the same distribution, and then averaged. Figure S10 shows the computed average transmittance spectra for various particle size polydispersities, σ_r. We see that the waveguide's performance is impaired by this polydispersity (presumably because of the affected waveguide edges), but unidirectional propagation is still observed for polydispersity values up to $\sigma_r = 0.020$.

B. Positional imperfections

Even in the absence of polydispersity, in self-assembled colloidal structures, the positions of individual particles deviate from their perfect lattice positions due to thermal effects. To probe the impact of these imperfections, we compute the transmittance properties of the waveguide structure shown in the main body of this work with the particle positions shifted randomly. The particles are placed with their position coordinates x and y drawn from a normal distribution centred on their respective ideal coordinates, (x_0, y_0) with the same standard deviation σ_p:

$$P(i) = \frac{1}{\sqrt{2\pi(\sigma_p)^2}} \exp\Big[-\frac{(i-i_0)^2}{2(\sigma_p)^2}\Big],$$

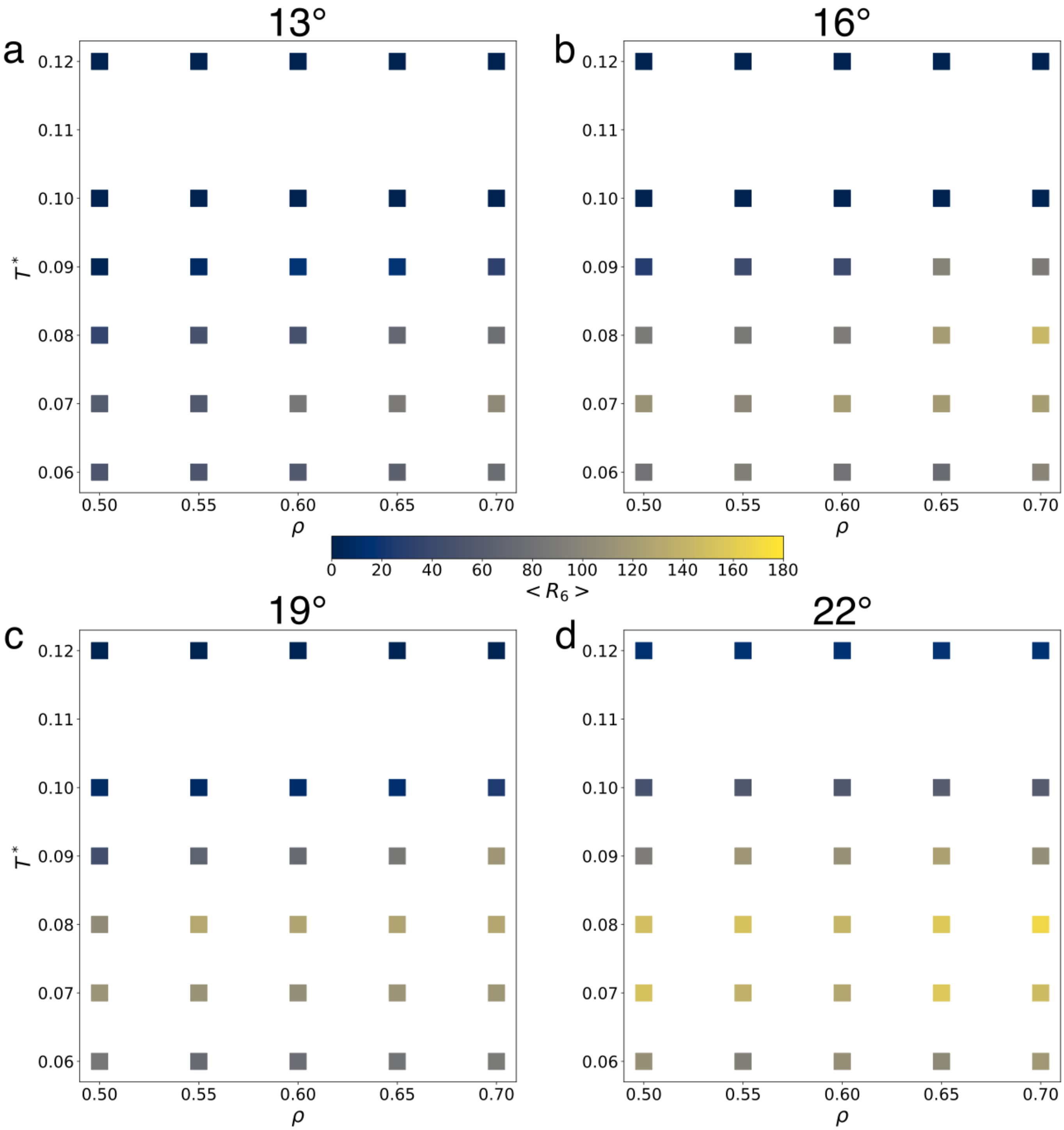

FIG. S6. Effects of patch size on the quality of the self-assembled honeycomb crystal. The results are for systems of 960 tetrahedral patchy particles in a 1:1 two-component mixture when subject to one-step cooling to various temperatures at different particle number densities from $T^* = 0.20$. (a,b,c,d) show the average number of 6-member rings identified in the self-assembled configurations in each simulation performed with the half-opening angles of 13°, 16°, 19°, and 22°, respectively. The central colour bar shows that darker colours correspond to fewer 6-member rings being present.

with i the new x or y coordinate and $i_0 = x_0$ or y_0. Transmittance spectra are averaged over 10 separate runs with different geometries but with positions drawn from the same distribution. Figure S11 shows the computed average transmittance spectra for waveguide structures with various levels of positional imperfection. We find that introducing these deviations from the perfect lattice compromises the waveguide's performance, again likely due to a disruption of

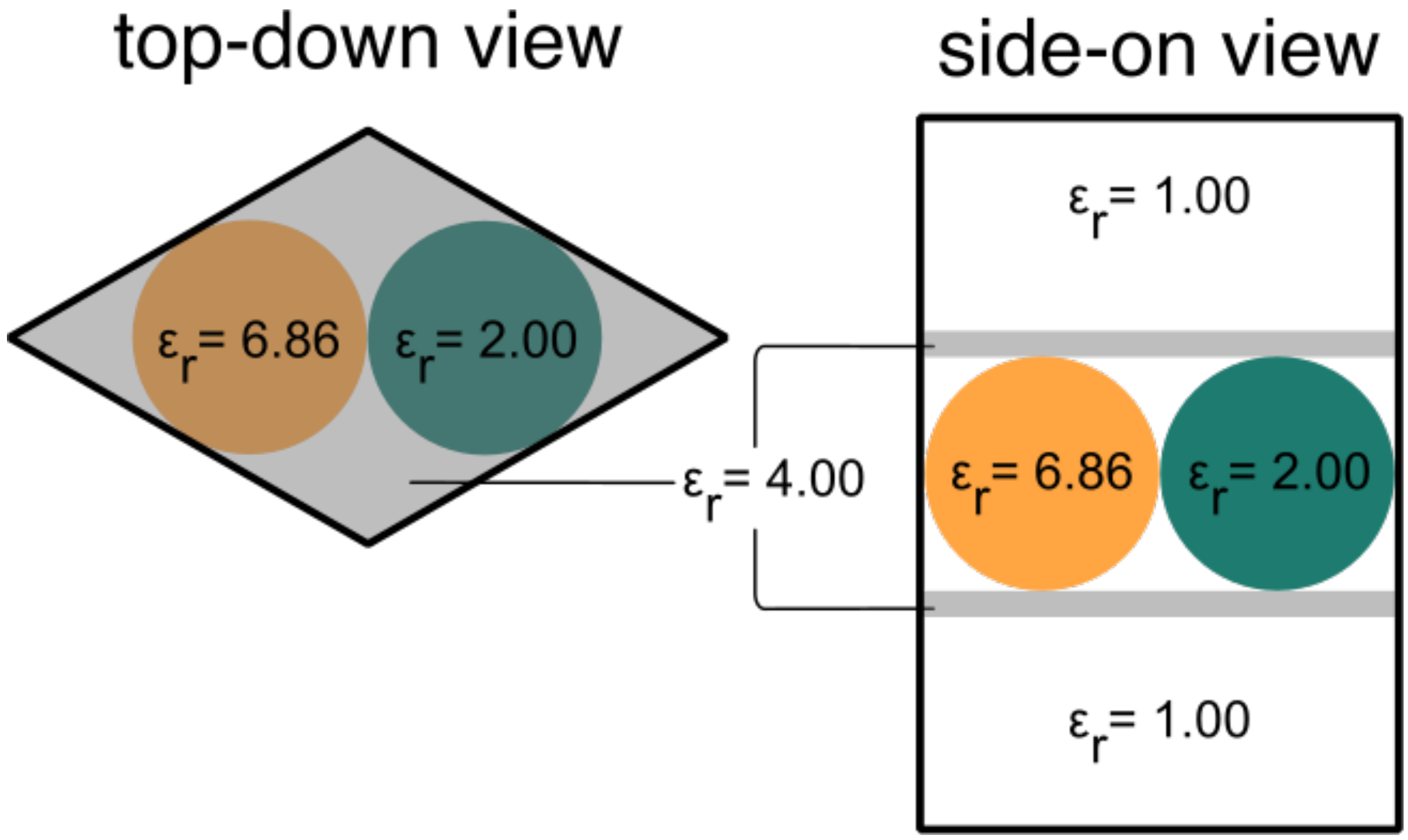

FIG. S7. Schematic of the unit cell used for band structure calculations, solid black lines indicate periodic boundaries. Air gaps ($\epsilon_r = 1.00$) are added above and below the slab containing the honeycomb unit cell to ensure there is no coupling between periodic layers.

symmetry at the waveguide edges. Despite this reduction in performance, we still observe unidirectional propagation within a narrower frequency range for σ_p values up to 0.150.

C. Polydispersity and positional imperfections

We also investigate the robustness of the unidirectional waveguiding to the combined effects of polydispersity and positional imperfection. Figure S12 shows that the behaviour is preserved even for values of $\sigma_r = 0.01$ and $\sigma_p = 0.075$. We note that the lower tolerance for particle size polydispersity represents less of a challenge since state-of-the-art synthetic techniques can routinely produce monodisperse colloidal suspensions.[S4] Conversely, colloidal self-assembly is susceptible to thermal effects, invariably leading to a degree of imperfections for the assembled structures relative to the perfect lattice. The tolerance towards these positional imperfections exhibited by the honeycomb crystal structure is thus encouraging for scalable fabrication of topological photonic devices for future applications.

VII. ROBUSTNESS OF DESIGN RULES TO INTERACTION SPECIFICITY

The robustness of our design rules for ring selection during self-assembly with respect to interaction specificity is discussed in the main body of the article, with reference to Fig. S13.

VIII. NATURE OF THE MELTING TRANSITION

We note in the main body of the manuscript that the self-assembly proceeds with a continuous decrease in the average energy per particle in both systems. We also observe a gradual evolution of ring statistics in the two systems, indicating a continuous ordering of the systems upon cooling, rather than a sharp first-order transition. When identical parameters are used for a preliminary investigation of melting, the average energy per particle for either system is observed to increase gradually, as shown in Fig. S14. In this context, it should be noted that system size is an important consideration for conclusive evidence of the nature of phase transitions in either system.

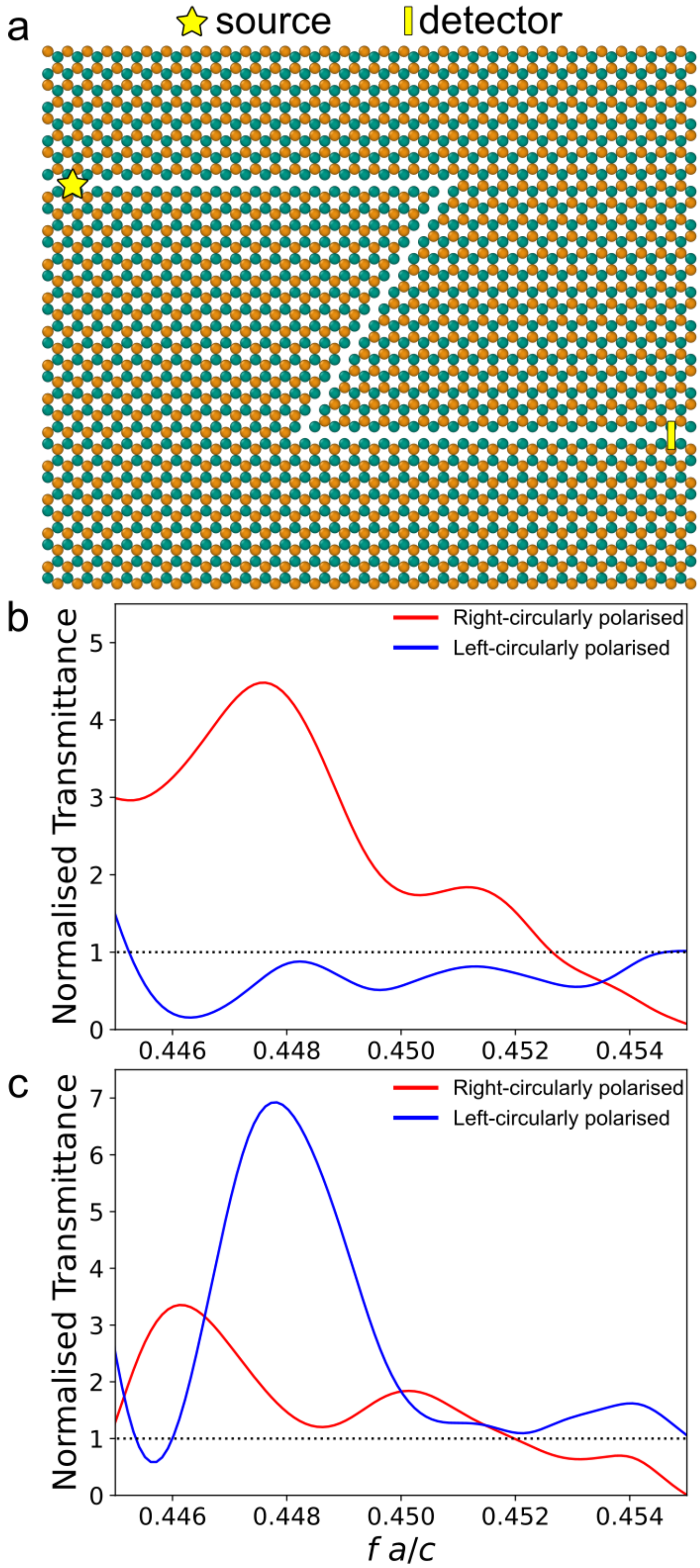

FIG. S8. **Unidirectionality in a Z-shaped waveguide.** (a) Top-down schematic of the waveguide structure constructed from two complementary structures, shifted with respect to one another to create a Z-shaped bend in the waveguide. The chiral light source and detector are placed on opposite sides of the waveguide. (b) Transmittance spectra for right- and left-circularly polarised light (RCP and LCP, respectively) through the waveguide when the source and detector are placed in the positions shown in (a). (c) Transmittance spectra for RCP and LCP through the waveguide when the source and detector swap their positions. Transmittance plots are normalised with respect to an empty slab structure; two dielectric layers with no honeycomb slab between them. Despite the sharp bends in the waveguide structure, we still observe unidirectional propagation, with RCP propagating to the right and LCP to the left.

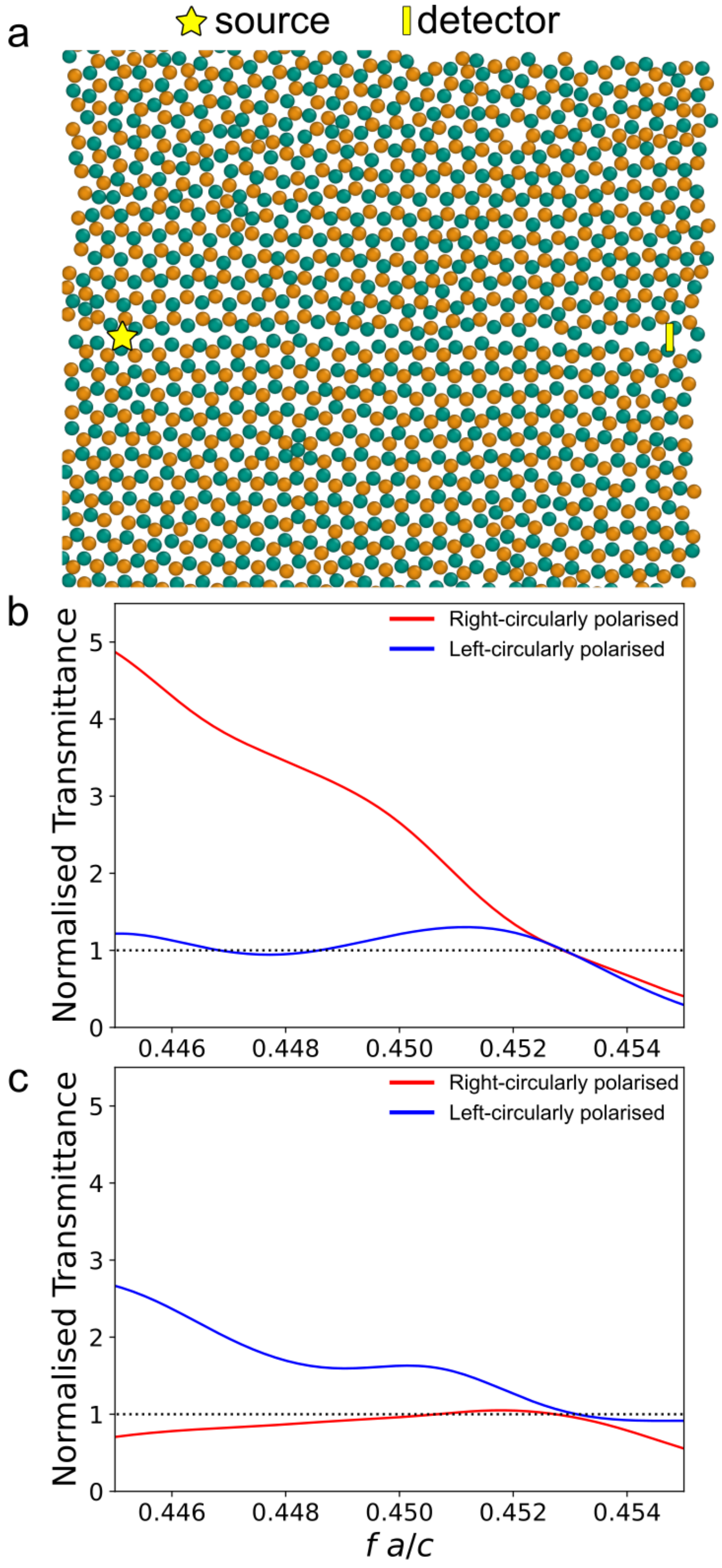

FIG. S9. **Unidirectionality in a waveguide, using self-assembled configurations.** (a) Top-down schematic of the waveguide structure constructed from two self-assembled crystallites with defects as sampled by Monte Carlo simulations. The chiral light source and detector are placed on opposite sides of the waveguide. (b) Transmittance spectra for right- and left-circularly polarised light (RCP and LCP, respectively) through the waveguide when the source and detector are placed in the positions shown in (a). (c) Transmittance spectra for RCP and LCP through the waveguide when the source and detector swap their positions. Transmittance plots are normalised with respect to an empty slab structure: two dielectric layers with no honeycomb slab between them. Despite having defects in this waveguide structure, we still observe a signature of unidirectional propagation, with RCP propagating to the right and LCP to the left.

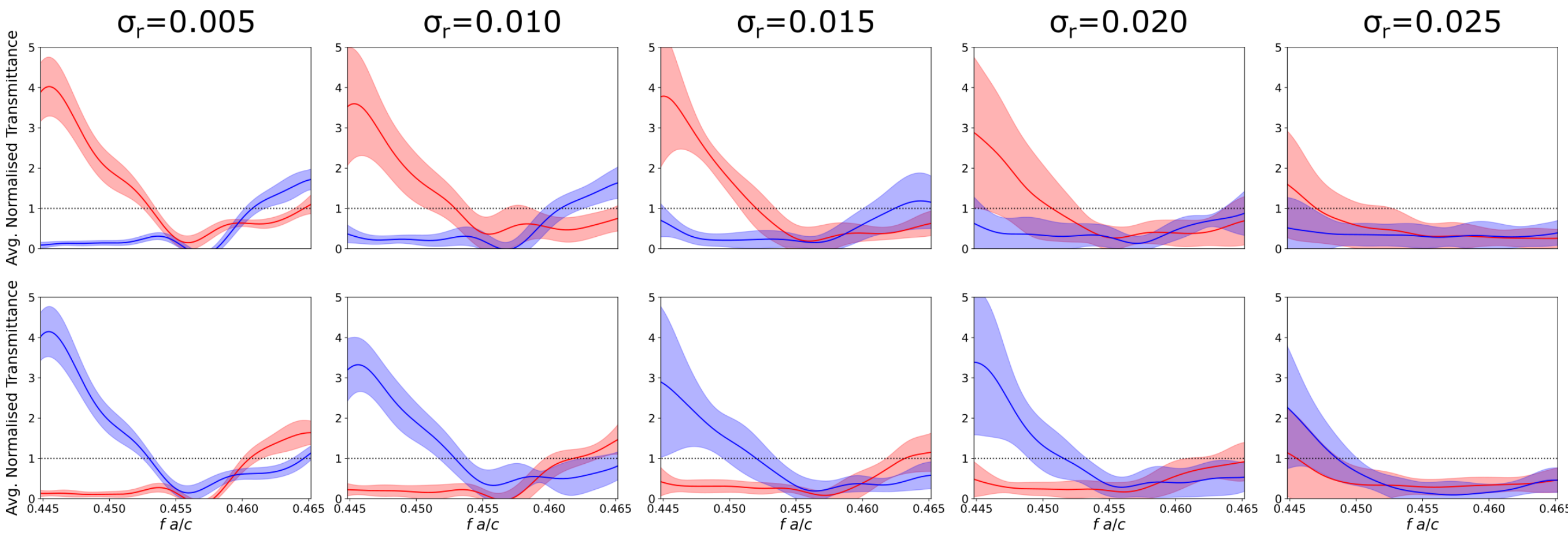

FIG. S10. Transmittance spectra for various particle size polydispersities, σ_r. Top row: averaged transmittance spectra for right- and left-circularly polarised light (red and blue lines, respectively) through the waveguide when the detector is placed to the right of the source at the opposite end of the waveguide. Bottom row: averaged transmittance spectra for right- and left-circularly polarised light (red and blue lines, respectively) through the waveguide when the source and detector positions are swapped. Transmittance values are shown relative to what is obtained for an empty slab structure (two dielectric layers with no honeycomb slab between them), indicated by the dotted line. Shaded regions show the standard deviation in the 10 transmittance spectra used to compute the average in each case.

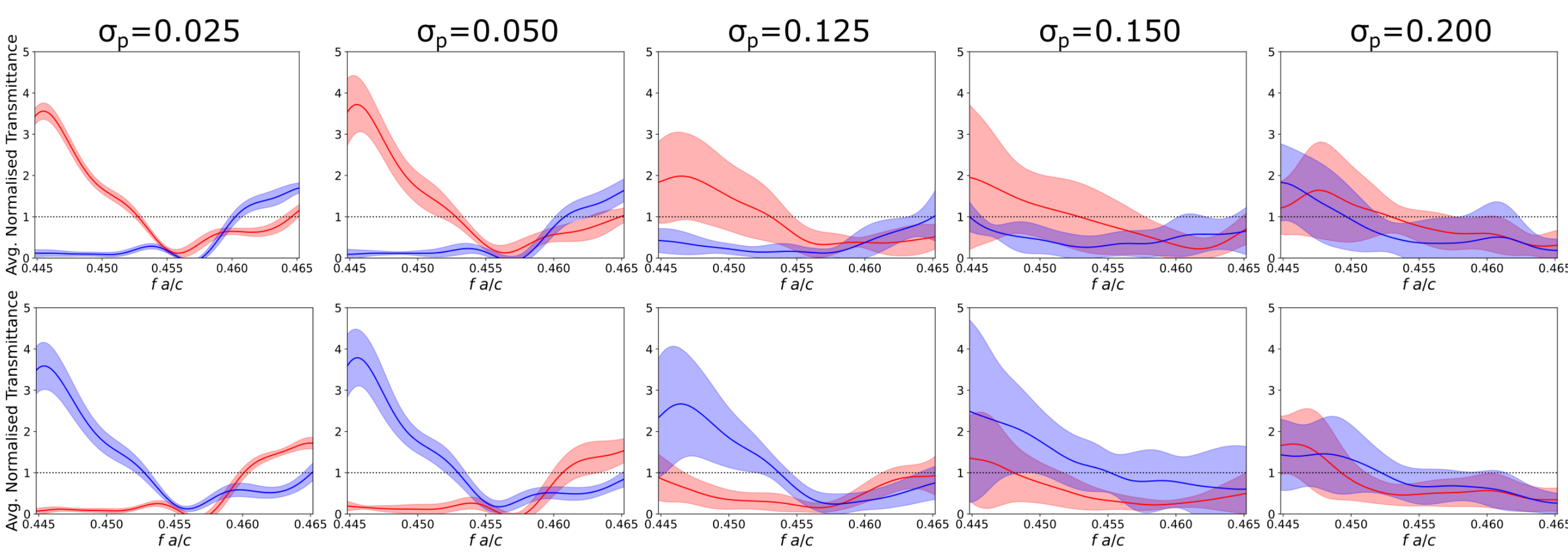

FIG. S11. Average transmittance spectra for waveguide structures with various levels of positional imperfection to simulate thermal noise. Here, the level of positional imperfection is characterised by the deviation from the perfect lattice location with a standard standard deviation, σ_p, as defined in the text of Supplementary Information. Top row: average transmittance spectra for right- and left-circularly polarised light (red and blue lines respectively) through the waveguide when the detector is placed to the right of the source at the opposite end of the waveguide. Bottom row: average transmittance spectra for right- and left-circularly polarised light (red and blue lines respectively) through the waveguide when the source and detector positions are swapped. Transmittance values are shown relative to what is obtained for an empty slab structure (two dielectric layers with no honeycomb slab between them), indicated by the dotted line. Shaded regions show the standard deviation in the 10 transmittance spectra used to compute the average in each case.

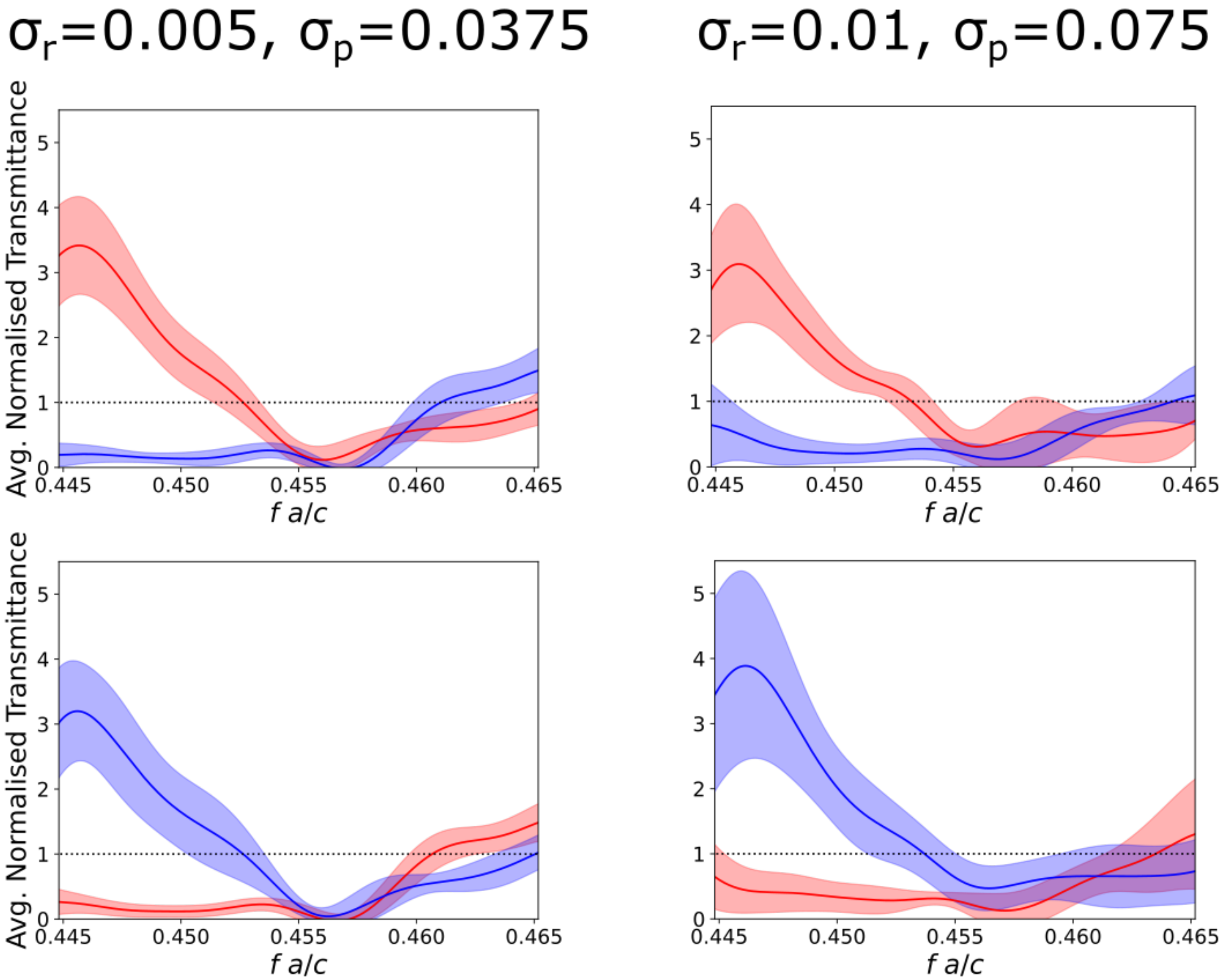

FIG. S12. Average transmittance spectra for waveguide structures with various levels of polydispersity, σ_r, and positional imperfection, σ_p. Top row: average transmittance spectra for right- and left-circularly polarised light (red and blue lines, respectively) through the waveguide when the detector is placed to the right of the source at the opposite end of the waveguide. Bottom row: average transmittance spectra for right- and left-circularly polarised light (red and blue lines, respectively) through the waveguide when the source and detector positions are swapped. Transmittance values are shown relative to what is obtained for an empty slab structure (two dielectric layers with no honeycomb slab between them), indicated by the dotted line. Shaded regions show the standard deviation in the 10 transmittance spectra used to compute the average in each case.

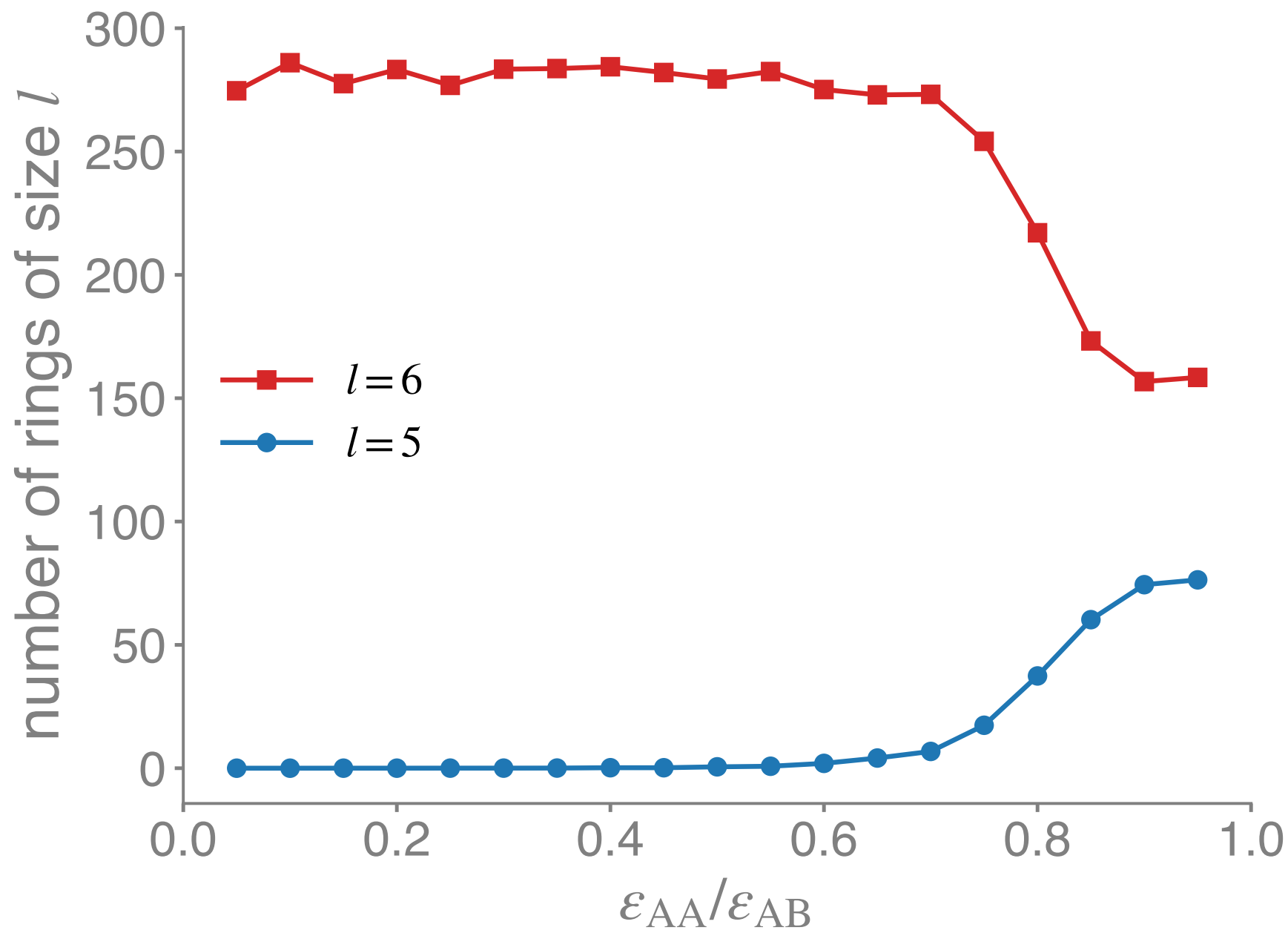

FIG. S13. **Robustness of ring-selection rules to the interaction specificity.** Number of 5- and 6-member rings as a function of the well-depth for interactions between species of the same type relative to that of different types ($\varepsilon_{AA}/\varepsilon_{AB}$) for systems of $N = 960$ tetrahedral patchy particles on a substrate at a reduced temperature of $T^* = 0.08$.

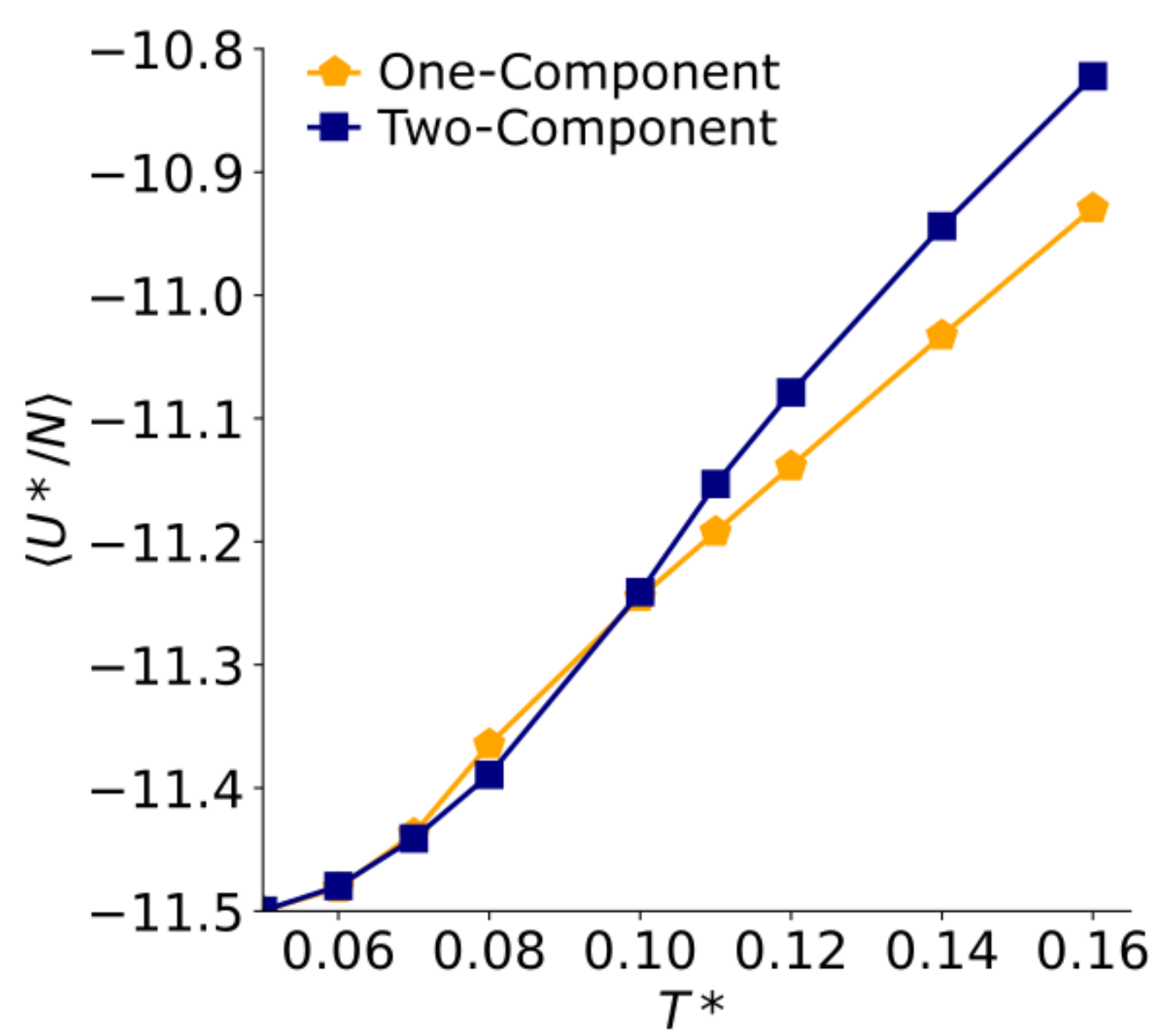

FIG. S14. **Melting in one-component and two-component systems of tetrahedral patchy particles on a substrate.** Average energy per particle for one-component and two-component systems of tetrahedral patchy particles on a substrate upon heating from a perfect honeycomb crystal. In both cases, melting begins around $T^* = 0.06$.

[S1] N. Kern and D. Frenkel, Fluid–fluid coexistence in colloidal systems with short-ranged strongly directional attraction, J. Chem. Phys. **118**, 9882 (2003).

[S2] P. J. M. Swinkels, Z. Gong, S. Sacanna, E. G. Noya, and P. Schall, Visualizing defect dynamics by assembling the colloidal graphene lattice, Nat. Commun. **14**, 1524 (2023).

[S3] J. D. Morrow, C. Ugwumadu, D. A. Drabold, S. R. Elliott, A. L. Goodwin, and V. L. Deringer, Understanding Defects in Amorphous Silicon with Million-Atom Simulations and Machine Learning, Angew. Chem. **136**, e202403842 (2024).

[S4] Y.-J. Kim, J.-B. Moon, H. Hwang, Y. S. Kim, and G.-R. Yi, Advances in Colloidal Building Blocks: Toward Patchy Colloidal Clusters, Adv. Mater. **35**, 2203045 (2023).
